# Advantageous Microwave-Assisted Suzuki Polycondensation for the Synthesis of Aniline-Fluorene Alternate Copolymers as Molecular Model with Solvent Sensing Properties

**DOI:** 10.3390/polym10020215

**Published:** 2018-02-22

**Authors:** Rebeca Vázquez-Guilló, Alberto Falco, M. José Martínez-Tomé, C. Reyes Mateo, María Antonia Herrero, Ester Vázquez, Ricardo Mallavia

**Affiliations:** 1 Instituto de Biología Molecular y Celular, Universidad Miguel Hernández, E-03202 Elche, Spain; rebeca.vazquez@goumh.umh.es (R.V.-G.); alber.falco@umh.es (A.F.); mj.martinez@umh.es (M.J.M.-T.); rmateo@umh.es (C.R.M.); 2 Departamento de Química Inorgánica, Orgánica and Bioquímica, Facultad Ciencias y Tecnologías Químicas, Universidad Castilla La Mancha, E-13071 Ciudad Real, Spain; se.mlcu@orerreh.ainotnaairam (M.A.H.); se.mlcu@zeuqzav.retse (E.V.); 3 Instituto Regional de Investigación Científica Aplicada (IRICA), Edificio Marie Curie, Universidad Castilla La Mancha, E-13071 Ciudad Real, Spain

**Keywords:** microwave-assisted, suzuki coupling, poly(fluorene-aniline), solvatochromic effect

## Abstract

Polymerization via Suzuki coupling under microwave (µW) irradiation has been studied for the synthesis of poly{1,4-(2/3-aminobenzene)-*alt*-2,7-(9,9-dihexylfluorene)} (**PAF**), chosen as molecular model. Briefly, µW-assisted procedures accelerated by two orders of magnitude the time required when using classical polymerization processes, and the production yield was increased (>95%). In contrast, although the sizes of the polymers that were obtained by non-conventional heating reactions were reproducible and adequate for most applications, with this methodology the molecular weight of final polymers were not increased with respect to conventional heating. Asymmetric orientation of the amine group within the monomer and the assignments of each dyad or regioregularity, whose values ranged from 38% to 95% with this molecule, were analysed using common NMR spectroscopic data. Additionally, the synthesis of a new cationic polyelectrolyte, poly{1,4-(2/3-aminobenzene)-*co*-*alt*-2,7-[9,9´-bis(6’’-*N*,*N*,*N*-trimethylammonium-hexyl)fluorene]} dibromide (**PAFAm**), from poly{1,4-(2/3-aminobenzene)-*co*-*alt*-2,7-[9,9´-bis(6’’-bromohexyl)fluorene]} (**PAFBr**) by using previously optimized conditions for µW-assisted heating procedures was reported. Finally, the characterization of the final products from these batches showed unkown interesting solvatochromic properties of the **PAF** molecule. The study of the solvatochromism phenomena, which was investigated as a function of the polarity of the solvents, showed a well-defined Lippert correlation, indicating that the emission shift observed in **PAF** might be due to its interaction with surrounding environment. Proven high sensitivity to changes of its environment makes **PAF** a promising candidate of sensing applications.

## 1. Introduction

Palladium coupling reactions, and, particularly, Suzuki polycondensations enable the synthesis of conjugated copolymers with interesting properties as efficient emitters [1,2,3,4,5,6,7,8,9,10,11,12,13,14,15,16,17,18,19]. In general terms, some of the advantages that are related to these reactions are: the need of only a small controlled catalytic amount of palladium catalyst; soft reaction conditions; great tolerance for a variety of functional groups; no generation of toxic products; and, no perturbation by the presence of water [20,21,22]. In particular, unlike direct arylation reactions in which numerous crosslinks occur, the Suzuki coupling reaction allows for obtaining homopolymers and copolymers of fluorene without defects [23,24,25,26,27,28,29]. Despite the great development of this reaction in the recent years and its many advantages over other methodologies, the synthesis of conjugated polymers using this type of polycondensation procedures requires further research to reduce their duration and energy consumption, for instance. In this sense, it is necessary to study new models and procedures to deeply understand these processes [30,31,32].

Synthesis of polyfluorenes, by Suzuki coupling reactions with conventional procedures, usually takes more than 48 h to produce polymers with convenient molecular weights as shown, for instance, in previous works from our lab [33,34]. Reaction times reduction is, therefore, one of the main targets in the optimization of the synthesis conditions [35,36,37,38,39].

Additionally, it is known that the balance between properties, such as the solubility and the effective length of the chains plays an important role in the interaction with other biomolecules and further applications. Thus, controlling the Suzuki polycondensation of novel conjugated polymers with defined properties, such as the effective length of the conjugated polymer chains, with narrow polydispersity (PDI) values and short reaction times, has been an important objective of previous works [21].

For this purpose, new sources of energy may open new opportunities. In recent years, the application of microwaves (µW) in organic synthesis has grown exponentially mainly due to drastic reaction time reductions [40,41,42], greater control of the reaction conditions [43,44] and improvement of the reproducibility [45], and, in many cases, increase of the reaction yields [46]. Currently, besides its use for the synthesis of simple organic molecules, the application of µWs in polymerization reactions is increasing [47,48,49]. In particular, the production of conjugated polymers by µW-assisted coupling reactions is becoming more popular, mainly because it shows a clear economic advantage over conventional heating [48]. For example, the polymerization that is achieved via Suzuki coupling by employing a µW-assisted process for the synthesis of poly(9,9-dihexylfluorene) reduced to 15 min of the reaction time required, once the conditions were optimized [30].

Consequently, the emergence of a new low-cost and quick process conforms an interesting scenario to prepare new copolymers à la carte. Furthermore, post-modifications with the adequate associated functionalities play an important role in the design of new conjugated polymers with applications in cross-disciplinary areas, such as chemistry, material sciences, or biological sciences. In this sense, one of the most common functionalization strategy is the addition of a charged group (either cationic or anionic) within their side-chains in order to obtain conjugated polyelectrolytes (CPEs). Moreover, it is also possible to obtain polyelectrolytes by incorporating pyridinium salts in the polymer backbone due to the reactivity of the amino group and its high donor effect [50].

**Figure 1:**
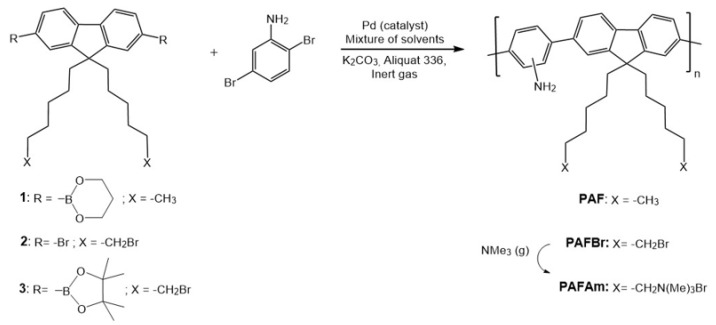
Suzuki coupling of poly-{1,4-(2/3-amino)benzene-*co*-*alt*-[9,9-bis(6´-X-hexyl)-2,7-fluorene]} derivatives (**PAF**, X = –CH_3_; **PAFBr**, X = –CH_2_Br and **PAFAm**, X = –CH_2_NMe_3_ Br).

Thus, in the present work, some aspects governing the order of the monomers in the polymer during the synthesis process have been also studied; in particular, the appearance in the process of an asymmetric monomer, which produces asymmetry in the polymer backbone. The orientation adopted by the asymmetric monomer defines the regioregularity (RR) of the final polymer. The degree of RR informs about the proportion of HT/TH (head to tail or tail to head) dyads when compared to HH (head to head) and TT (tail to tail) dyads may be tested. A common example of non-regiosymmetic conjugated polymer is poly(3-hexylthiohene-2,5-diyl) (P3HT), in which the reactivity of the asymmetric monomer generates non-equivalent dyads that can be quantified by ^1^H NMR [51]. Assignments of these dyads in copolymers based on fluorene and aniline unities were proposed by Yamaguchi et al. [50,52]. Based on these assumptions, we tried to understand the processes involved in the copolymerization of commercial diboronate 9,9-dihexylfluorene and an asymmetric monomer, 2,5-dibromoaniline, via Suzuki coupling using microwave irradiation. In other words, the generation of poly[1,4-(2/3-aminobenzene)-*alt*-2,7-(9,9-dihexylfluorene)] (**PAF**), as the new asymmetric model is proposed here to optimize the parameters appointed before by using non-conventional heating. In this work, this procedure has been also used to develop a synthetic route in the preparation of a novel trimethylammonium polyelectrolyte based on diboronate 9,9-di(6-bromohexyl)fluorene and 2,5-dibromoaniline as precursor monomers (Figure 1).

Finally, in addition to the mentioned-above alternative synthesis procedure, the photophysical properties of **PAF** have been characterized, showing that this polymer displays an interesting solvatochromic effect on its emission profile that can extend the potential applications of this kind of CPEs.

## 2. Materials and Methods

### 2.1. Materials

All of the solvents used for chemical reactions were HPLC grade. 2,5-Dibromoaniline, 9,9-dihexylfluorene-2,7-diboronic acid bis(1,3-propanediol)ester (**1**), trioctylmethylammonium chloride (Aliquat 366), tetrakis(triphenylphosphane) palladium(0) [Pd(PPh_3_)_4_], 4-[bis(2-methyl-2-propanyl)phosphane]-N,N-dimethylanilinedichloropalladium(2+) [Pd(Amphos)_2_Cl_2_] and dichloro[1,1′-bis-(diphenylphosphanyl)ferrocene]palladium(2+) *cis-*[PdCl_2_ dppf] were obtained from Sigma-Aldrich Co. (Madrid, Spain).

### 2.2. Instrumentation

*Microwave equipment.* µW-assisted polymerizations were performed by using a CEM Discover reactor in 10 mL standard Pyrex vessels. The temperature and power was monitored during the process with a calibrated infrared temperature control and pressure sensors. The rest of the parameters were selected and modified, as described in corresponding results section for optimization purposes.

*Size Exclusion Chromatography (SEC).* SEC analysis was carried out on Shimadzu LC-20AD (Shimadzu, Kyoto, Japan) with an index refraction detector RID-10A (Shimadzu, Kyoto, Japan) and an Evaporative Light Scattering Detector ELSD 3300 (Alltech Associates Inc., Deerfield, IL, USA). SEC analysis of 20 μL of samples were injected in a column Plgel 5 μm MIXED-C; 2 × (300 × 7.5 mm i.d.) from Polymer Laboratories Ltd. (Salop, UK). Samples around 3–5 mg/mL in THF (as eluent) and filtered through nylon 0.45 micron membranes. SEC data were initially calibrated using Polymer Laboratories EasiCal Polystyrene (PS) standards.

*Nuclear Magnetic Resonance and Fourier Transform Infrared Spectroscopy.*^1^H and ^13^C NMR spectra were recorded on a Bruker AVANCE 500 spectrometer (Bruker, Ettlingen, Germany), with tetramethylsilane as an internal reference. Processing of spectra was done using TopSpin 3.2 (Bruker, Rheinstetten, Germany). FTIR spectra were obtained using Bruker IFS66s model spectrometer (Bruker, Karlsruhe, Germany) with samples prepared as KBr pellets.

*Absorption and Fluorescence Measurements.* Absorption spectra of the polymers were recorded in different solvents using a Shimadzu 1673 spectrophotometer (Shimadzu, Kyoto, Japan). Emission spectra were recorded in a PTI QuantaMaster-4 spectrofluorometer (Photon Technology International, Lawrenceville, GA, USA) at 90° detection angles. Excitation and emission wavelengths are auto-calibrated and selected by means of a computer-controlled FeliX32TM Software package (Photon Technology International, Lawrenceville, GA, USA). Corrected steady state fluorescence emission was performed using low absorption solutions.

*Thermal and Thermogravimetric analysis.* Thermal analyses were performed using differential scanning calorimetry (DSC) in a Perkin Elmer Pyris model 6 apparatus. Quantities of 2–4 mg of samples were scanned for a range of temperatures from 30 to 300 °C under nitrogen atmosphere at 20 °C/min rate (three heating/cooling cycles). Glass transition temperature (*T*g) was recorded on the second heating curve. Thermogravimetric analyses (TGA) were performed by using a TGA Q50 (TA Instruments, New Castle, DE, USA) and data was recorded under nitrogen atmosphere by equilibrating at 100 °C, followed by a slope increasing at 10 °C/min up to 800 °C.

### 2.3. Synthesis and Characterization

*Monomer Synthesis.* 2,7-Dibromo-9,9’-bis(6’’-bromohexyl)fluorene (**2**) and bis[9,9’-bis(6’’-bromohexyl)fluorenyl]-4,5,5,5-tetramethyl[1.3.2]dioxaborolane (**3**) were obtained according to previous procedures [53].

*General Polymerization.* In a round-bottom flask or µW reactor vessel, for conventional or µW-assisted heating procedures, respectively, containing a magnetic stir bar, 0.5 mmol of each monomer and the corresponding catalyst percentage (which varied depending on the reaction) were added in the presence of 2 mL of potassium carbonate (2.5 M) and dissolved in a mixture of solvent (either toluene/water or THF/water, depending on the reaction) and one drop of Aliquat 366. Each mixture was stirred and degassed under argon atmosphere during the polymerization processes. Then, the solvent was evaporated and the residue was dissolved in chloroform and re-precipitated in methanol twice. Finally, the polymer was dried under vacuum at 40 °C, obtaining a light-brown or beige solid.

*Poly[1,4-(2/3-aminobenzene)-co-alt-2,7-(9,9´-dihexylfluorene)]* (**PAF**). Equimolar monomers 2,5-dibromoaniline and 9,9-dihexylfluorene-2,7-diboronic acid bis(1,3-propanediol)ester (**1**).

^1^H NMR (500 MHz, CDCl_3_, ppm): δ = 7.86–7.60 (br m, 4H, Ar), 7.54 (br s, 2H, Ar), 7.38–7.33 (br d, 1H, Ar), 7.19–7.21 (br d, 1H, Ar), 7.13 (br s,1H, Ar), 4.24–3.89 (br s, 2H, NH_2_), 2.05 (m, 4H, CH_2_(1)), 1.20–1.05 (m, 12H, CH_2_(2, 4 and 5)) and 0.85–0.70 (br m, 10H, CH_2_(3) and CH_3_), (see Figures S1–S3).

^13^C NMR (125 MHz, CDCl_3_, ppm): δ = 14.2 (CH_3_), 22.7 (CH_2_(2)), 24.1 (CH_2_(3)), 29.9 (CH_2_(5)), 31.7 (CH_2_(4)), 40.5 (CH_2_(1)), 55.4 (C Fluorene), 114.5 (CH_Ar_), 118.0 (CH_Ar_), 120.1 (CH_Ar_), 120.2 (CH_Ar_), 121.7 (CH_Ar_), 123.8 (CH_Ar_), 126.1 (CH_Ar_), 127.3 (CH_Ar_), 127.9 (CH_Ar_), 131.1 (CH_Ar_), 132.5 (C_Ar_), 138.0 (C_Ar_), 140.0 (C_Ar_), 141.0 (C_Ar_), 142.2 (C_Ar_), 144.1 (C_Ar_), 151.7 (C_Ar_) and 151.9 (C_Ar_), (see Figures S4–S6).

IR (KBr disc, cm^−1^): *3462*, *3380* (NH_2_), 3026, 2925, 2852, *1610* (NH), 1460, 1307, 1246, 809, 754, 696 and 546 cm^−1^.

*Poly{1,4-(2/3-aminobenzene)-co-alt-2,7-[9,9´-bis(6’’-bromohexyl)fluorene]}* (**PAFBr**). Equimolar monomers 2,5-dibromoaniline and bis[9,9’-bis(6’’-bromohexyl)fluorenyl]-4,5,5,5-tetramethyl[1.3.2]-dioxaborolane (**3**), using the best non-conventional conditions of **PAF**, yield a beige solid 86 % SEC (THF, and PS standards): *M*w: 5825 g/mol; PDI: 2.0.

^1^H NMR (500 MHz, CDCl_3_, ppm): δ = 7.87–7.56 (br m, 4H, Ar), 7.51 (br s, 2H, Ar), 7.38–7.33 (br d, 1H, Ar), 7.21–7.02 (br s, 2H, Ar), 4.24–3.89 (br s, 2H, NH_2_), 3.27 (br t, 4H, CH_2_–Br), 2.04 (m, 4H, CH_2_(1)), 1.66 (br s, 4H, CH_2_(5)), 1.32-1.05 (m, 8H, CH_2_(3 and 4)) and 0.90–0.60 (m, 4H, CH_2_(2)), (see Figure S7).

^13^C NMR (125 MHz, CDCl_3,_ ppm): δ = 23.9 (CH_2_(2)), 25.1 (CH_2_(3)), 29.4 (CH_2_(5)), 32.9 (CH_2_(4)), 34.1 (CH_2_–Br), 40.3 (CH_2_(1)), 55.3 (C Fluorene), 114.5 (CH_Ar_), 118.6 (CH_Ar_), 120.4 (CH_Ar_), 120.6 (CH_Ar_), 121.8 (CH_Ar_), 123.9 (CH_Ar_), 126.2 (CH_Ar_), 127.4 (CH_Ar_), 128.9 (CH_Ar_), 131.2 (C_Ar_), 138.1 (C_Ar_), 140.0 (C_Ar_), 140.4 (C_Ar_), 143.9 (C_Ar_), 144.1 (C_Ar_) and 151.4 (C_Ar_), (see Figure S8).

IR (KBr disc, cm^−1^): *3450, 3378* (NH_2_), 2928, 2853, *1610* (NH), 1556, 1461, 1352, 1256, 1145, 809, 755, 695, 642 (–CH_2_–Br) and 557 cm^−1^.

*Poly{1,4-(2/3-aminobenzene)-co-alt-2,7-[9,9´-bis(6’’-N,N,N-trimethylammoniumhexyl)fluorene]} dibromide* (**PAFAm**). Quaternization of **PAFBr** by treatment with trimethylammine, following previous reported studies in our lab, yield 80% [54].

^1^H NMR (500 MHz, DMSO-d_6_, ppm): δ = 7.87–7.05 (br m, 9H, Ar), 4.24–3.80 (br s, 2H, NH_2_), 3.21 (br s, 4H, CH_2_–N), 3.03 (br s, 18H, N^+^CH_3_), 2.10 (br S, 4H, CH_2_(1)), 1.52 (br s, 4H, CH_2_(5)), 1.15 (br m, 8H, CH_2_(3 and 4)) and 0.72 (br s, 4H, CH_2_(2)), (see Figure S9).

^13^C NMR (125 MHz, DMSO-d_6,_ ppm) Data obtained by HMQC (Figure S10): δ = 21.9 (CH_2_(2)), 23.3 (CH_2_(3)), 25.5 (CH_2_(4)), 28.5 (CH_2_(5)), 30.7 (CH_2_(1)), 52.0 (CH_3_–N), 65.0 (CH_2_–N), 110.0 (CH_Ar_), 1140.4 (CH_Ar_), 118.9 (CH_Ar_), 120.4 (CH_Ar_), 123.1 (CH_Ar_), 125.8 (CH_Ar_), 126.7 (CH_Ar_), 127.3 (CH_Ar_), 129.0 (CH_Ar_) and 130.7 (CH_Ar_). (Quaternary carbons were insignificant and negligible detectable signals).

IR (KBr disc, cm^−1^): *3405 (broad +NR_4_)*, 3023, 2928, 2855, 1562, 1462, 1400, 1260, 1024, 965, 909, 814, 754, 698 and 547 cm^−1^.

## 3. Results and Discussion

### 3.1. Optimization of the Synthesis of **PAF** and Characterization of the Different Batches

#### 3.1.1. General Considerations

The polymerization batches of the molecular model **PAF**, via Suzuki coupling, were obtained by using equimolar concentrations of monomers, in a mixture of solvents with K_2_CO_3_, as base, and Aliquat 366, as phase transfer agent (Figure 1). A total amount of 28 batches were synthesized under different conditions. As a standard procedure, the yield and the color of the final product, as well as the molecular weight distribution of the polymers and the RR of their amine groups in the final copolymer were studied. As a summary in general terms, obtained reaction yields were between 34% and 99%, molecular weights between 3.54 and 36.49 kg/mol and degrees of RR between 38% and 95%. Macroscopically, it was observed that bright yellowish colors usually corresponded to the solids of batches with lower molecular weights or yields, while batches with higher molecular weights or yields showed darker brown colors. All of the polymers were soluble in common organic solvents, such as tetrahydrofuran (THF), chloroform, toluene, and, even, *N*,*N*-dimethylformamide (DMF), with no evidence of aggregation or gel formation. 

#### 3.1.2. Characterization of **PAF** Batches

The molecular weight distributions of synthesized polymers were measured by SEC with polystyrene calibration (all collected data is compiled in Figures S11 and S12 of supporting data). In fluorene-based copolymers, SEC analysis overestimate the molecular weights and show multiple well-defined peaks in some batches, possibly due to the formation of different oligomers [34,55].

IR spectra showed identical characteristic peaks that were assignable to stretching vibration ν(NH_2_) at 3462 and 3380 cm^−1^, and corresponding in-plane blending of (N–H) at 1610 cm^−1^. They were also found to be very similar to the spectrum of polymer without the amine group, *poly[1,4-benzene-co-alt-2,7-(9,9´-dihexylfluorene)]* (PBF) (see supplementary information, Figure S13). 

In this work, thermal properties, like glass transition (*T*g) and decomposition temperature recorded at 5% mass loss (T5) were studied in order to detect differences among selected batches. **PAF** *T*g was around 180 ± 5 °C and it was difficult to obtain in a second cycle of heating. T5 was around 325±15 °C in all batches, while the range of mass loss at final temperature (800 °C, inert atmosphere) was 55–65% (see supporting information, Figure S14). 

Absorption and emission spectra of **PAF** in chloroform solutions from several batches were virtually identical with absorption and emission maximum wavelengths at 360 and 426 nm, respectively (Figure 6A and Table 3). The most significant characteristic of the **PAF** emission spectra was the absence of a shoulder or second peak, a common event in similar fluorene copolymers, such as PBF. A clear red-shift in the emission spectra was observed when changing from chloroform to other solvents, what it will be further described later on in this work.

#### 3.1.3. Study of the Regioregularity of Synthesized **PAF** by ^1^H-NMR

The presence of an asymmetric amino group in each aniline monomer within the molecular structure of **PAF** made the study of its RR possible. Depending on the position of the amino groups during the first step of the catalytic cycle (oxidative addition), there can be coupled four types of units or dyads: tail-to-tail (TT), head-to-head (HH), head-to-tail (HT), and tail-to-head (TH). These positions are statistically possible during a growing chain, for further details see supplementary data (Figures S15 and S16).

**Figure 2:**
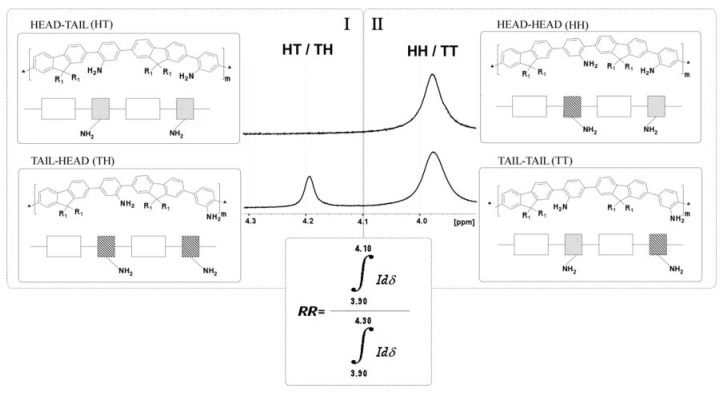
Enlarged region of ^1^H-NMR spectra (CDCl_3_, 500 MHz) corresponding to amine protons areas for two **PAFs** batches. Four dyads and formula of regioregularity (RR) in this proposed model.

Coupling bromoanilines with commercial boronate ester of fluorene was previously reported by Yamaguchi et al. [50] who showed ^1^H-NMR in CDCl_3_ data by means of the following models (supplementary data, Figure S17): 1a (3.82 ppm, HH) coupling with 2-bromoaniline; 1b (3.85 ppm TT) with 3-bromoaniline and 1c (4.00 ppm for HT or TH) coupling a mixture of 2- and 3-bromoaniline. However, in our spectra, only two groups of NH_2_ were mostly found when **PAF** batches were analysed in deuterated chloroform (Figure 2). Assuming Yamaguchi’s data, the new assignment of dyads in our polymers was HT or TH structures for chemical shift δ above 4.10 ppm (area I) and HH/TT for structures below δ 4.10 ppm (area II). In Figure 2, the separation between the two areas and their corresponding structures of the dyads, together with the formula that defines RR in this model, are shown.

In this work, the RR of each batch was defined and obtained by the integration of the amine protons area II (δ 4.10–3.90 ppm) with respect to the total integrated area in amine zone (δ 4.3–3.90 ppm), followed by its correction with the aliphatic area (at 2 ppm, 2x–CH_2_– adjacent to C9 fluorene), taken as reference. Some signals of low intensity that were detected close to these regions were attributed to probable non-completed polymerizations, which might have resulted in low molecular weight polymers, or oligomers and aniline monomers that did not react. The molar percentage ratio of integrated areas between both zones (indicated as % RR) estimates the number and position of the amine groups within the backbone as well as their assignments that could be used to understand changes in experimental conditions.

RR percentage might work as an indicator of the preferential orientation of the amine groups in the final polymer. In this study, the values ranged from 38% to 95%. Percentages of RR > 80%, less content in dyads of TH/HT respect to TT and/or HH dyads (Table 1, #2, #3, #8, and #9), means that then next coupling prefers again this dyad. In turn, RR percentages from 20% to 80% would meet a random process (Table 1, #1, #4, #5, #6, #7, and #10). Lower percentages (<20%) with large presence of TH and/or HT dyads would indicate a preferential block copolymer in the alignment of the amino groups (“hairstyles in the same direction”), this latter case is not found herein.

After synthetic procedures, a selection of representative batches were analysed by ^1^H-NMR (% RR) and SEC (*M*w) in order to establish correlations between both factors (see supplementary data Figure S12 and Table S1). For high and low RR, minor variations were found in aromatic and aliphatic proton regions (Figure S1), what it was confirmed by further analysing the structure of **PAF** by mono- and bi-dimensional NMR experiments (Figures S2–S6).

#### 3.1.4. Synthesis Optimization of **PAF**

In order to optimize polymer synthesis conditions, **PAF** batches that are produced by different procedures have been characterised and compared, paying special attention to the heating sources and catalysts employed. Most reactions under classical conditions were performed at 80 °C, while µW-assisted reactions were done at 135 °C. The high temperature achieved in µW-assisted procedures give us the opportunity to reduce the reaction time. Therefore, µW-assisted heating offers a simple and fast methodology to study and/or optimize the synthesis reactions, which allows for controlling with high precision the conditions of the polymerizations. Furthermore, Suzuki coupling cycle involves intermediate high polar or ionic species, which might be excellent candidates for µW energy transfer in order to obtain good yields [56,57]. Otherwise, although most of the reactions performed under conventional conditions were made using a conventional flask (at atmospheric pressure) and a reflux system, the oil bath reaction was also tested by using a pressure vessel, which allowed for working above the reflux temperature of the solvents and reaching temperatures that were comparable to those attained by µW irradiation. On the other hand, experiments with µW-assisted heating were performed here by using two different operational modes, which are provided by the instrument: the dynamic and the solid phase synthesis (SPS). The dynamic mode is an option with standard settings where the power is automatically modulated based on the maximum temperature it can get, while in the SPS mode, the irradiation cycles in bursts are performed by keeping the temperature with a prefixed deviation, 5 °C usually (see modes in supporting Figure S18). 

In general (Table 1), reaction rates were about two orders of magnitude faster by µW-assisted heating than by conventional heating methods at atmospheric pressure. By performing reactions above boiling temperature, synthesis production was also improved in some aspect in comparison to conventional heating methods. It is remarkable that high yields were obtained in shorter times in most of the batches by µW-assisted polymerization (around 20 min in batches #6 and #8, for instance) respect to conventional operation (#1, #2). At the same temperature, 135 °C, the use of pressure vessel (#3) leads to a noticeable yield of polymer with high general properties and without important differences in terms of RR respect to µW-assisted heating (#8), as previously described [58].

Temperature is a critical parameter in a chemical reaction; in this case, the temperature above the boiling point is determinant to accelerate the polymerization process. Furthermore, it looks like there might be a fine equilibrium between the reduction on reaction time and the degradation of some of the species involved in the process. However, polymerizations obtained by increasing the reaction temperature, and, thus accelerating the process, are comparable to longer ones, as observed in a previous work [34].

As indicated in Table 1, SPS operation cycle (#7, #8) achieved better results than dynamic mode (#5, #6), with excellent yields and good distributions of the polymer *M*ws (PDI values close to 2), as also noted for polyhomofluorenes irradied with the same µW instrument and similar reaction conditions [30]. For this reason, SPS mode was selected for further µW-assisted polymerization studies.

**Figure 3:**
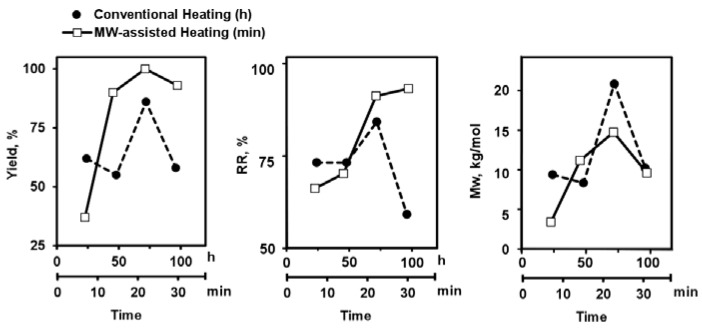
Effect of the time polymerization in microwave-assisted in SPS mode (µW-SPS) (empty squares) and classical heating (filled circles) at different temperatures (135 °C for µW-assisted and 80 °C for conventional heating) versus percentage of yield (**left**), regioregularity RR (**middle**) and weight-average molecular weigth, *M*w (**right**).

Subsequently, a time course of the polymerization procedure was performed by both heating processes (Figure 3). Previous results were confirmed from these assays, showing that conventional heating reaction done at atmospheric pressure is two orders of magnitude slower than the µW-assisted one in the SPS mode.

Another parameter, evaluated because of its relevance for synthesis procedures, was the use of different catalysts. In this sense, the best results (in terms of higher yields and *M*w values), from all tested polymerizations, were achieved for the palladium zero ([Pd(PPh_3_)_4_]) catalyst in comparison to palladium II ([Pd(Amphos)_2_Cl_2_] and *cis*-[PdCl_2_(dppf)]), catalysts that are commonly used in other synthesis models [34]. Therefore, the effect of different concentrations of palladium zero catalyst on both procedures was evaluated. In the first place, a gradient of concentrations of this catalyst was tested in both of the procedures and their final products characterized (Table 2).

Obtained yields were excelent in all cases (Minimum: 85% for #13; Maximum: 100% for #14). *M*ws of final polymers increased with the concentration when using classical heating procedures, but in non-conventional heating it was observed that an optimal result peacked at 3% of palladium zero (#7). Regarding RR, in classical heating, the number of coupled dyads TT/HH was substantially greater (#11, 93%) than in µW-assisted heating (#13, 84%). It is noticeable that RR tended to decrease with the amount of catalyst, independently of the heating process that is used. Efforts to systematically optimize the µW-assisted polymerization conditions to obtain **PAF** were studied, such as the solvent and volumes employed in the reaction vessels, as well as power and temperature changes in the SPS mode settings (see supplementary data for futher results in Tables S3–S5), were carried out. In general terms, the use of THF instead of toluene produces no differences on the yield but a great effect on the *M*w, with a considerable reduction in this value. Furthemore, a power of 150 W and temperature fluctuation (∆*T*) of 5 °C seems to be the optimal condition in terms of yield and *M*w.

**Figure 4:**
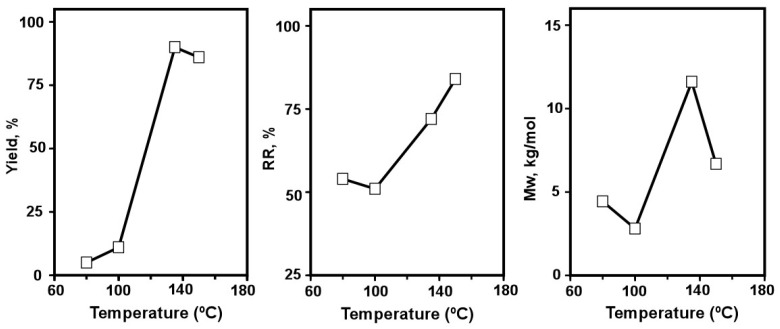
Temperature variation in microwave-assisted polymerizations (µW-SPS) respect to percentage of yield (**left**), regioregularity RR (**middle**), and weight-average molecular weigth, *M*w (**right**).

Finally, the effect of the final temperature in the polymerization reaction was also analysed by using SPS mode µW-assisted heating (Figure 4, and Table S5).

Figure 4 shows the progression of the yields, RR and *Mw*s over temperature until 150 °C, as it has been previuosly reported for homopolyfluorene, at about 150 °C there was an efficient polymerization with free-defect structures [30]. The optimal temperature for achieving the highest yield and *Mw* values is 135 °C, while the ratio of peaks of amino groups in NMR is high (almost 75%), but not the highest (84% at 150 °C). Remarkably, at 150 °C, the yield was similar to that obtained at 135 °C, RR was higher (84%), but *M*w decreased significantly (6.68 kg/mol respect to 11.60 kg/mol, value achieved at 135 °C). Regarding RR, by using this procedure, it was necessary to increase temperature over 100 °C in order to overcome 50% RR.

### 3.2. Synthesis of a New Cationic Polyelectrolyte (**PAFAm**)

To extend the potential applications of **PAF** to biochemical research, the cationic polyelectrolyte **PAFAm** was synthesised by transformation of neutral **PAFBr**. The synthetic route and structure of this polyelectrolyte is also shown in Figure 1. Polymer bromide, **PAFBr**, was obtained following the optimal conditions established in the previous **PAF** model (µW-assisted heating in SPS mode). Thus, the polymer was obtained in a 86% yield, with an average molecular weight of 5825 g/mol (PDI = 2.0), as determined by SEC with PS standard. The structure was confirmed by NMR data, with a broad triplet at 3.27 ppm with correspondence with carbon at 32.9 ppm (supplementary Figures S15 and S16).

**Figure 5:**
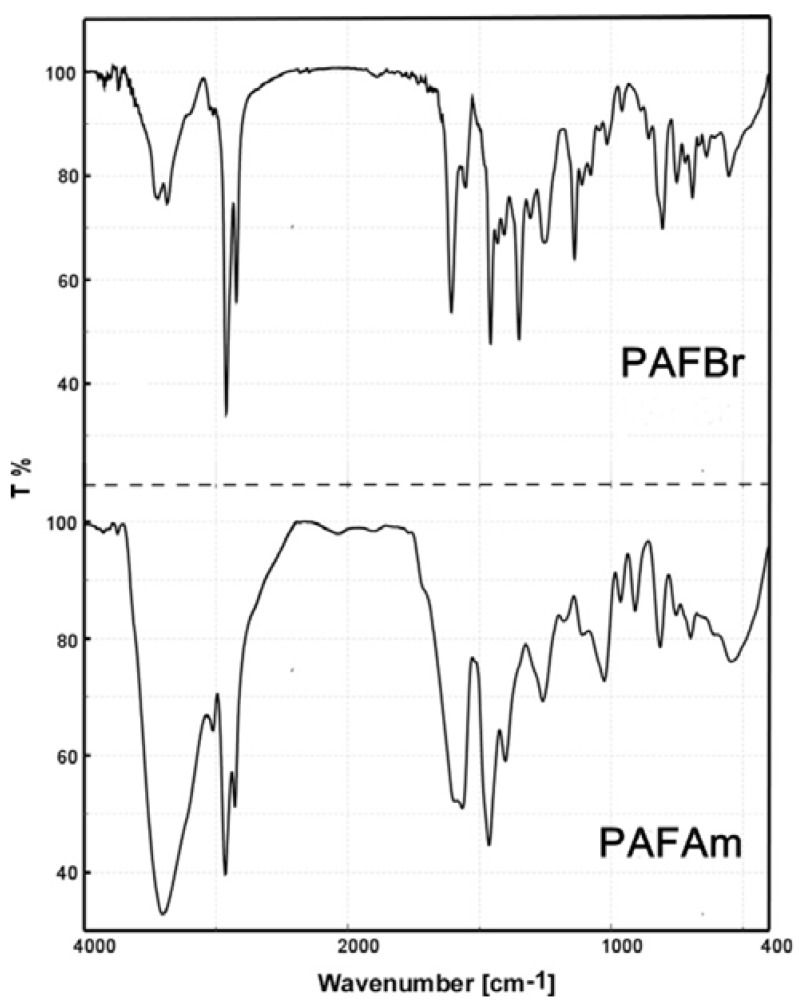
Comparative FT-IR spectra of new polymers synthesized **PAFBr** and **PAFAm,** in BrK pellets.

Figure 5 shows the IR spectrum of this new precursor polymer, **PAFBr**, which present the two frequencies of vibration of ν(NH_2_) amine groups at 3450 and 3378 cm^−1^, and a typical band assigned to the alkyl bromide –CH_2_Br (642 cm^−1^).

Quaternization of **PAFBr** was successfully obtained with total conversion (>97%) and a high purification yield (80%) of the new polyelectrolyte (**PAFAm**), using the treatment with trimethylamine, as previously reported [53,54]. Nucleophilic substitution was corroborated by proton and bi-dimensional HMQC NMR in DMSO-d_6_. Characteristic signals at 3.21 and 3.03 ppm, corresponding to –CH_2_N^+^(CH_3_)_3_ assignments and their carbons at 52 and 65 ppm, –CH_2_N^+^(CH_3_)_3_, respectively (supplementary Figures S9 and S10).

FT-IR spectra of **PAFAm** (bottom) and **PAFBr** (top) in BrK pellet was shown in Figure 5, where the disappearance of the band at 642 cm^−1^ and the appearance of an ammonium band at 3405 cm^−1^ covering the amine bands, can be observed. In complementary data, Figure S19 shows the electronic absorption and emission spectra of polymers **PAFBr** in chloroform and **PAFAm** in water solutions performed at room temperature. Both spectra present one maximum absorption at 345 and 352 nm, and their corresponding maximum emission bands, at 431 and 458 nm, respectively, a large Stokes shift (∆λ was 86 and 106 nm **PAFBr** and **PAFAm**), evidencing a solvent dependence.

### 3.3. Fluorescence Solvatochromism of **PAF**

**Figure 6:**
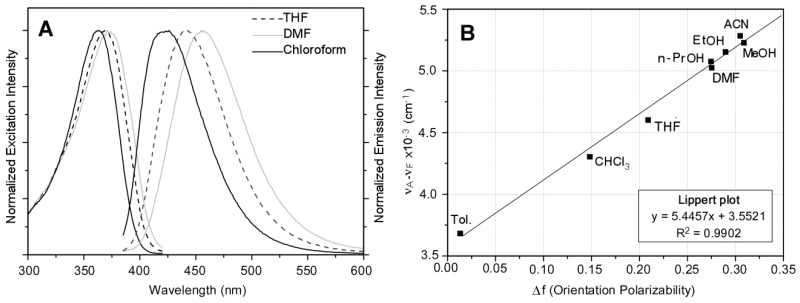
(**A**) Normalized fluorescence excitation and emission spectra of the same batch of **PAF** in several solvents (Abs. less 0.05 a.u.): chloroform (solid line), THF (dashed line) and DMF (dotted line). (**B**) Lippert plot or effect of solvent polarity on the Stokes´ shift of **PAF**.

Figure 6A shows red-shift in the emission spectra when changing from chloroform to other solvents, such as THF and DMF, and no increase in the vibronic structure was detected. In THF, the emission peak red-shifted around 20 nm, while in DMF, a shift of 30 nm was observed respect to chloroform. Absorption and excitation spectra were less sensitive to the solvent change than the emission spectrum, with red-shifts of 6 nm (THF) and 10 nm (DMF) respect to the value obtained in chloroform (Figure 6A). Due to the fact that these solvents show different polarity, the above-mentioned behavior suggests the possible existence of dipole-dipole interactions between **PAF** and solvent molecules [59]. 

In order to get more insight in this question, the absorption and fluorescence emission spectra of **PAF** were obtained in other solvents with different polarity. Table 3 shows the absorption and emission data for a constant concentration of **PAF** (1 μM; 0.07–0.04 a.u.) using eight different solvents. The absorption spectra displayed a weak solvatochromic effect, which suggests a small value for the ground state dipole moment of **PAF**. In contrast, the emission spectra showed an evident solvent dependence, with a clear bathochromic shift of the fluorescence maximum with increasing solvent polarity. The fact that a much larger solvatochromic effect is observed in the emission, when compared to the absorption spectrum, suggests that the dipole moments of **PAF** are significantly different between their ground and excited states. The excited state displays a larger dipole moment relative to the ground state, and, consequently, its stabilization is increased in a more polar environment [60].

The fluorescence solvatochromism of **PAF** was analyzed with the Lippert equation, which relates the Stokes shifts, in cm^−1^ ($\Delta \bar{\nu }={\bar{\nu }}_{\mathrm{abs}}^{\max}-{\bar{\nu }}_{\mathrm{fluo}}^{\max}$), of the fluorophore in different solvents to the orientation polarizability, ∆*f* (Table 3). This plot is shown in Figure 6B and was reasonably linear, supporting the hypothesis of dipole-dipole interactions between **PAF** and solvent molecules being mainly responsible for the bathochromic shift. No significant differences were found between protic and non-protic solvents, suggesting that hydrogen bonding of the amine group with the solvent is not responsible for this shift. Table 3 also displays the fluorescence quantum yield of **PAF** in different solvents. With exception of DMF, a decrease in this value was observed with increasing the polarizability of the solvent, which could suggest that the excited state structure of **PAF** varies with the solvent [61]. This behavior could be also attributed to the low solubility of **PAF** in polar solvents and the subsequent formation of polymer aggregates, which led to lower emission intensities due to an increase in the interchain energy transport mechanism.

Some commercial solvatochromic fluorophores, such as 2,6-ANS (6-anilinonaphthalene-2-sulphonic acid), 1,8-ANS (8-anilinonaphthalene-1-sulphonic acid), Prodan or ADMAN, (6-derivates of 1,2-dimethyaminonaphathelene), exhibit emission properties that are highly sensitive to their immediate environment and have been extensively used for investigating macromolecular interactions, protein conformational changes, or membrane phase transition detection [59]. The solvatochromic properties of **PAF**, not found in similar fluorene copolymers, could be used to detect this type of biochemical process as well as for other sensing applications.

## 4. Conclusions

In conclusion, we have investigated the µW-assisted polymerization via Suzuki coupling of **PAF** model, which contains alternated aniline and fluorene units. By this methodology, a significant reduction in time was achieved in comparison to classical conditions. This novel and fast methodology gives excelent yields and moderate average *Mw*s (adequate for some applications), and keeps inalterated the physicochemical behaviour of the products. This methodoly has been successfully set-up for obtaining a new cationic polyelectrolyte.

The characterization of the final polymer revealed two interesting features. On one hand, ^1^H-NMR data allowed for exploring the asymmetric orientation of the amine groups (regioregularity) in polyfluorenes, establishing a correlation between the assignments of the dyads. Our results suggest that the reaction mechanism has preferences for the activation of a position, thus the high regioregularity found is caused by preferential coupling of TT and/or HH dyads, which will condition the following coupling to go to the same dyad. On the other hand, the **PAF** model has been found to show a strong non-specific solvatochromic effect, what might be employed as an environmental probe in biochemical applications. This will be the scope of further works.

## Figures and Tables

**Table 1:** Effect of different heating reaction modes and catalysts on the polymerization of **PAF** ^a^.

	Catalyst	Heating conditions			Yield (%)	*M*w ^c^ (kg/mol)	PDI ^d^	n ^d^	% RR by NMR ^e^
1	[Pd(PPh_3_)_4_]	Oil bath;conventional flask	80 °C	24 h	62	9.90	1.9	22	75
2				72 h	86	20.77	1.6	47	87
3		Oil bath;cressure vessel	135 °C	22 min	72	6.39	2.1	14	83
4				24 h	34	3.54	1.5	8	78
5		µW;Dynamicmode^b^	135 °C	14 min	57	6.91	2.0	16	70
6				22 min	73	11.80	1.9	27	74
7		µW;SPS mode ^b^	135 °C	14 min	90	11.60	2.0	26	73
8				22 min	99	15.02	2.1	34	94
9	[Pd(Amphos)_2_Cl_2_]			14 min	71	5.43	1.9	12	86
10	*cis*-[PdCl_2_(dppf)]			14 min	47	6.26	1.8	14	64

^a^ All polymerizations were carried out with 3 % of catalyst in Toluene/H_2_O (2:1) *v*/*v*. ^b^ µW-assisted reactions were carried out at 150 W in either Dynamic or solid phase synthesis (SPS) mode, the latest with ∆*T* = 5 °C (see supporting Figure S18). ^c^ *M*w = weight-average molecular weight, estimated by SEC in THF on basis polystyrene calibration. ^d^ PDI (Polydispersity index) = *M*w*/M*n; where *M*n = number-average molecular weight, and n (number of monomer unities) = *M*w*/M*n; where *M*u = molecular weight unity (441.7 g/mol). ^e^ Percentages of regioregularity (% RR) were calculated in based on ratio NMR to integrated the Area II (3.9 > δ > 4.1 ppm) over Total Area (3.9 > δ > 4.3 ppm), see Figure 2. Relative error 4%.

**Table 2:** Effect of the concentration of the palladium zero catalyst on the polymerization of **PAF**.

#	Heating Mode	[Pd(PPh_3_)_4_] (%)	Yield (%)	*M*w ^c^ (kg/mol)	PDI ^d^	*n*^d^	% RR by NMR ^e^
11		1	90	10.70	1.9	24	93
2	Oil bath ^a^	3	86	20.77	1.6	47	87
12		6	86	36.49	2.7	83	86
13		1	85	5.73	1.7	13	84
7	µW SPS mode ^b^	3	90	11.60	2.0	26	72
14		6	100	6.45	1.6	15	69

^a^ Conventional polymerizations at atmospheric pressure were carried out at 80 °C, 72 h; Toluene/H_2_O (2:1) *v*/*v*. ^b^ µW-assisted were carried out at 135 °C, 14 min; 150 W and ∆*T* = 5 °C; Toluene/H_2_O (2:1) *v*/*v*. ^c,d,e^ Same description on Table 1.

**Table 3:** Optical properties of **PAF** in several solvents.

Solvent	∆*f* ^a^	λ_A_^max^(nm)	λ_E_^max^(nm)	ν_A_ − ν_E_(cm^−1^)	Ф_PL_^b^
Toluene	0.0132	363	419	3682	0.50 ± 0.05
Chloroform	0.1482	360	426	4303	0.49 ± 0.06
THF	0.2089	366	443	4601	0.55 ± 0.05
n-Propanol	0.2746	361	442	5076	0.18 ± 0.02
DMF	0.2754	371	456	5024	0.54 ± 0.03
Ethanol	0.2898	362	445	5152	0.16 ± 0.02
Acetonitrile	0.3050	357	440	5284	0.11 ± 0.02
Methanol	0.3089	363	448	5227	0.10 ± 0.02

^a^ Polarizability ∆*f* = (ε – 1/2ε + 1) – (*n*^2^ − 1/2*n*^2^ + 1) Lippert equation, where ε is dielectric constant and *n* is refractive index using data from Handbook of chemistry and physic [62]. ^b^ Quantum yield was calculated by triplicate probes, comparing the emission to that of a standard solution of quinine sulphate in sulphuric acid 0.1 M (Φ_PL_ = 0.546) at 25 °C [63].

## Supplementary Materials

The following are available online at http://www.mdpi.com/2073-4360/10/2/215/s1, which contains experimental details, SEC chromatograms mono- and bi-dimensional NMR spectra and additional figures and tables.
