# Supplementary material for: Advantageous Microwave-Assisted Suzuki Polycondensation for the Synthesis of Aniline-Fluorene Alternate Copolymers as Molecular Model with Solvent Sensing Properties
Source: Polymers (Basel). 2018 Feb 22;10(2):215. doi: 10.3390/polym10020215 (PMC6415332; doi:10.3390/polym10020215)
Supplement: Supplementary file 1 [file polymers-10-00215-s001.pdf]

Advantageous microwave-assisted Suzuki polycondensation for the synthesis of aniline-fluorene alternate copolymers as molecular model with solvent sensing properties

Rebeca Vazquez-Guillo[a]*, Alberto Falco*[a]*, M. José Martinez-Tome* [a]*, C. Reyes Mateo*[a]*, Maria Antonia Herrero*[b,c]*, Ester Vazquez*[b,c] *and Ricardo Mallavia**[a]

Supplementary Data, Summary:

- Pag. 2-7: NMR spectra of PAF, PAFBr and PAFAm.
- Pag. 8-11: GPC Chromatograms of batches PAF.
- Pag. 12: FTIR and TGA
- Pag. 13-15: Proposed mechanism and details.
- Pag. 16: Modes of μW-assisted polymerization and electronic spectra of PAFBr and PAFAm.
- Pag. 17-18: Tables of μW -assisted of PAF.

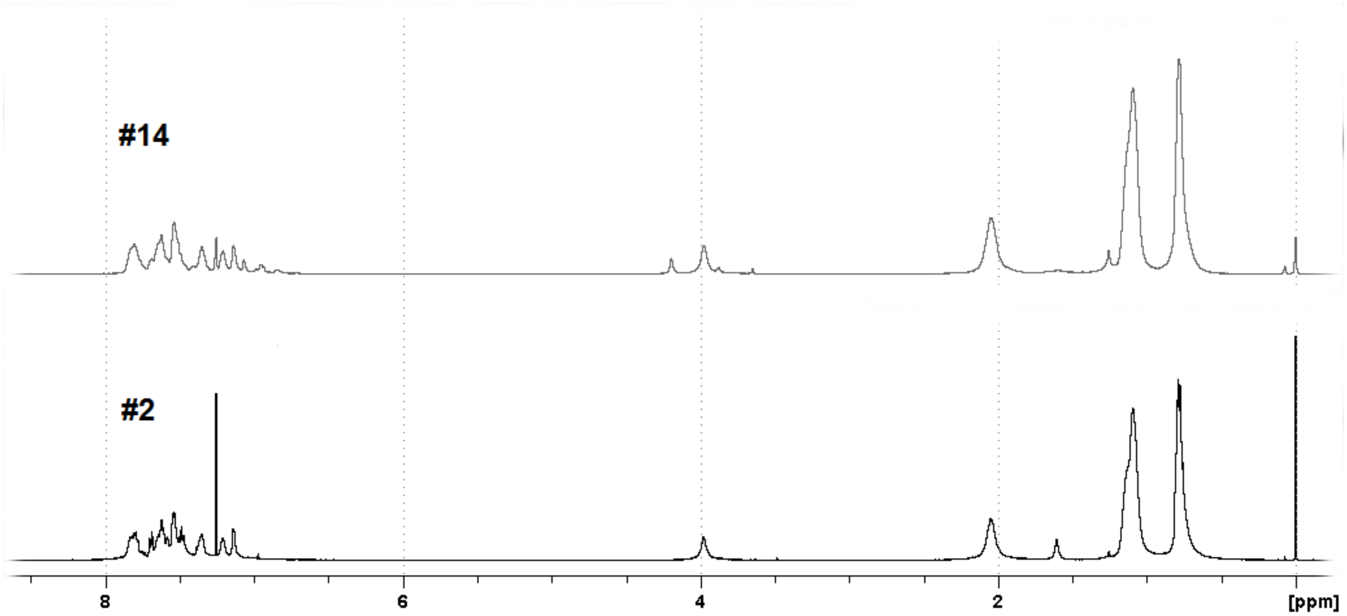

Figure S1. ^{1}H-NMR spectra comparatives of **PAF** batches (#14, %RR=69, Mw: 6450kg/mol; and #2, %RR=87%, Mw: 20775kg/mol) in $CDCl_3$, 500MHz.

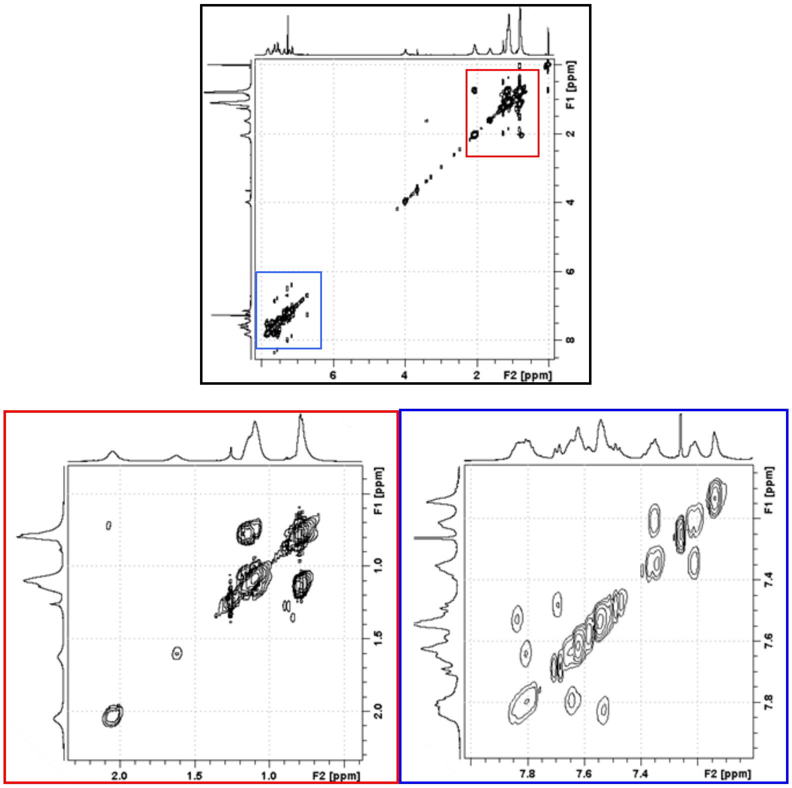

Figure S2. 2D ^{1}H-^{1}H **COSY** NMR spectra of **PAF** (top). Aliphatic (red) and aromatic (blue) zones.

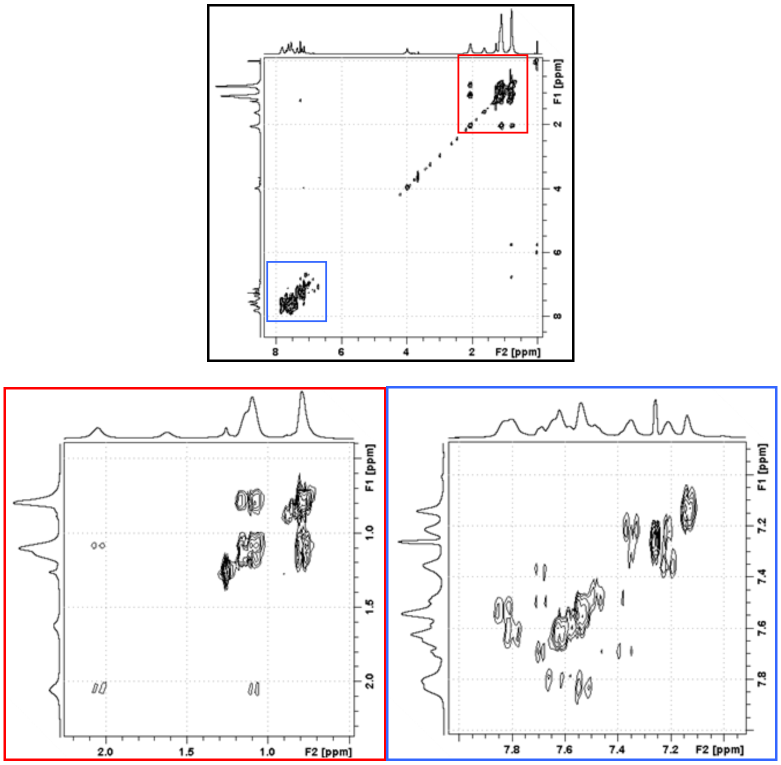

Figure S3. 2D ^{1}H-^{1}H **TOCSY** NMR spectra of **PAF** (top). Aliphatic (red) and aromatic (blue) zones.

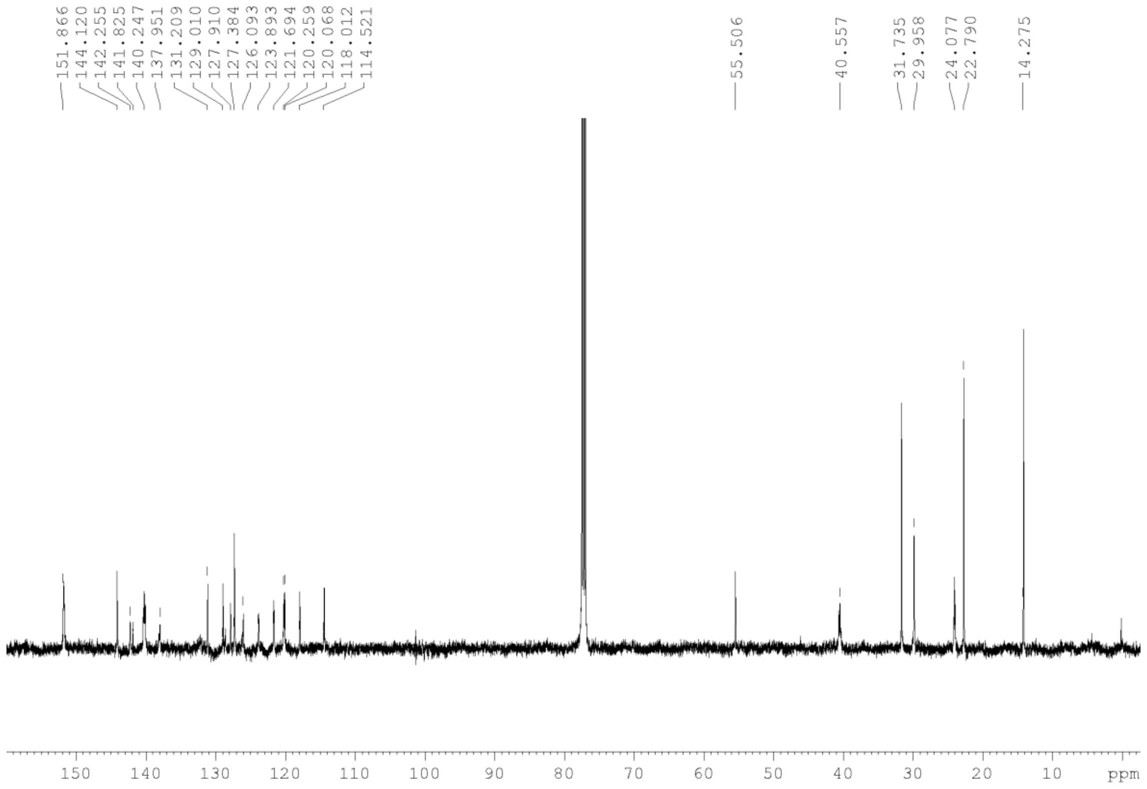

Figure S4. ^{13}C-NMR spectrum of **PAF** in $CDCl_3$, 125 MHz.

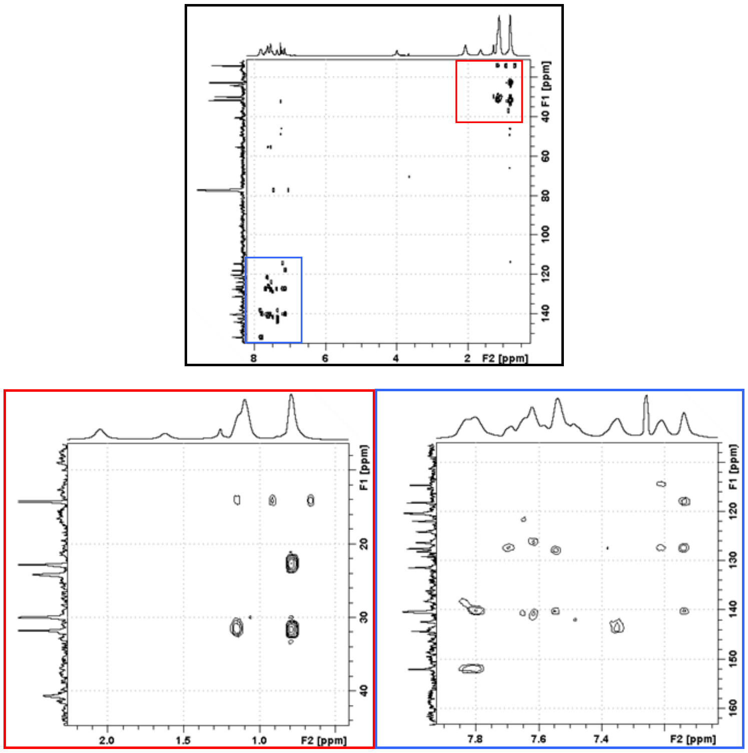

Figure S5. 2D ^{1}H-^{13}C **HMBC** NMR spectra of **PAF** (top). Aliphatic (red) and aromatic (blue) zones.

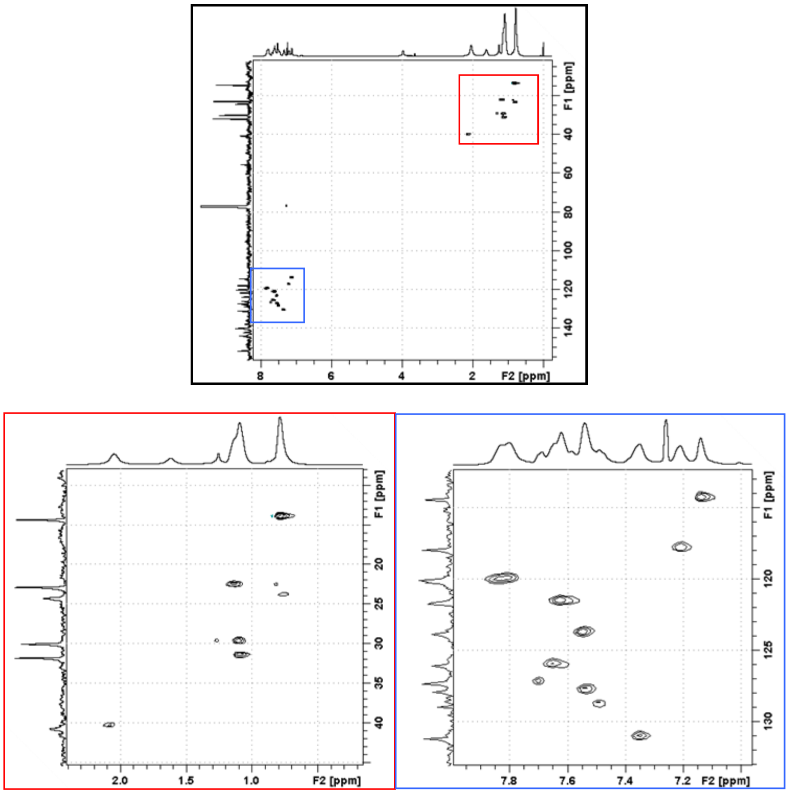

Figure S6. 2D ^{1}H-^{13}C **HMQC** NMR spectra of **PAF** (top). Aliphatic (red) and aromatic (blue) zones.

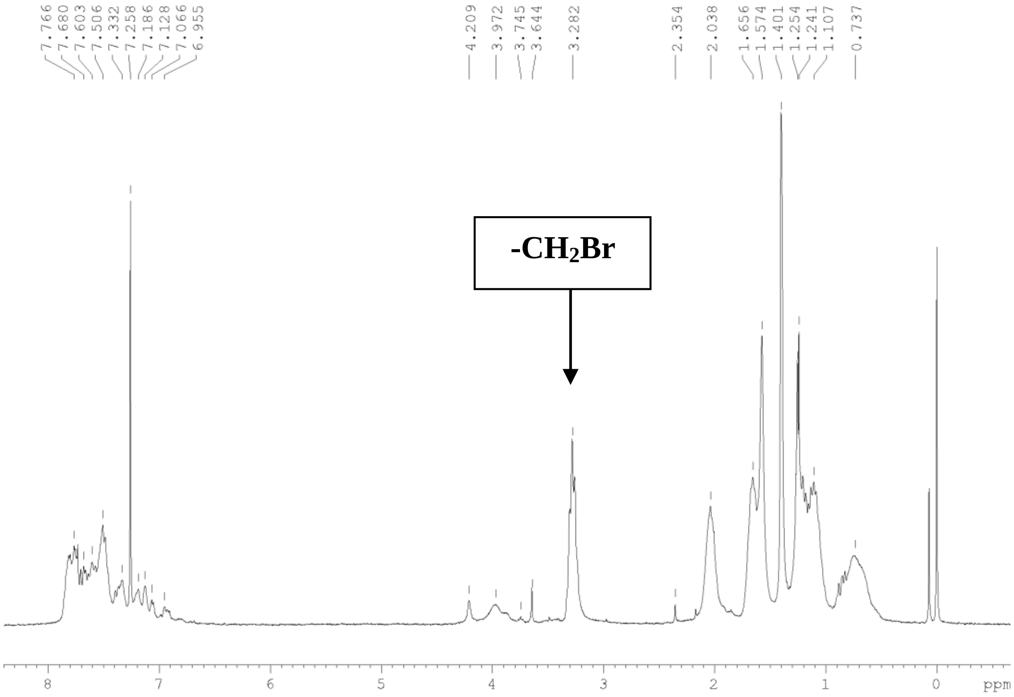

Figure S7. ^{1}H-NMR spectrum of **PAFBr** in $CDCl_3$.

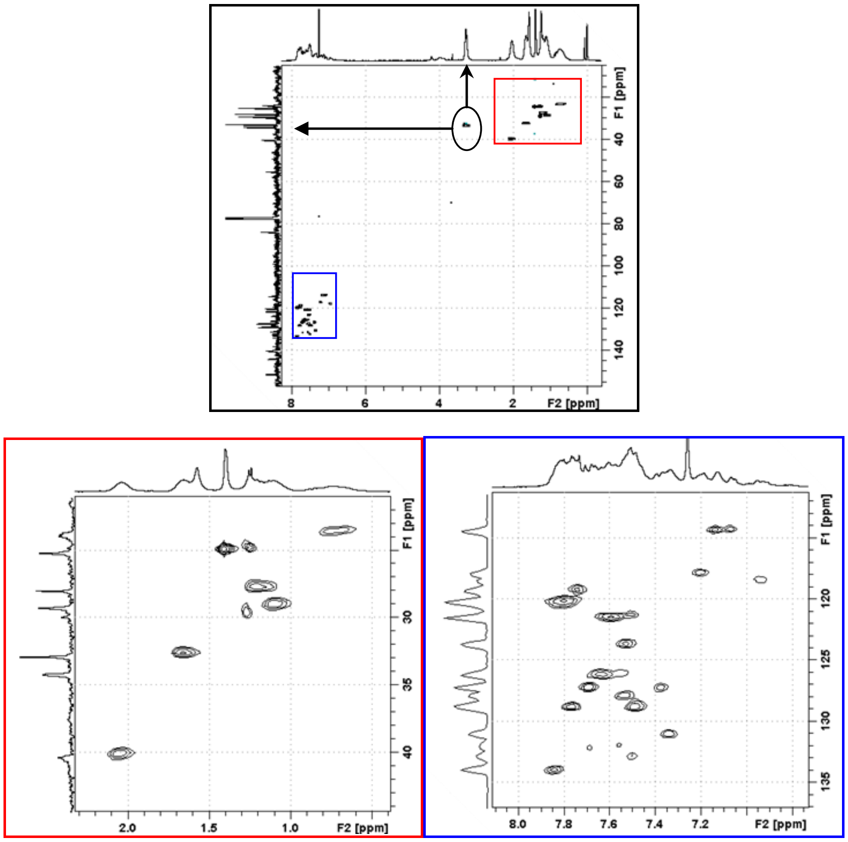

Figure S8. 2D ^{1}H-^{13}C **HMQC** NMR spectra of **PAFBr** in $CDCl_3$.

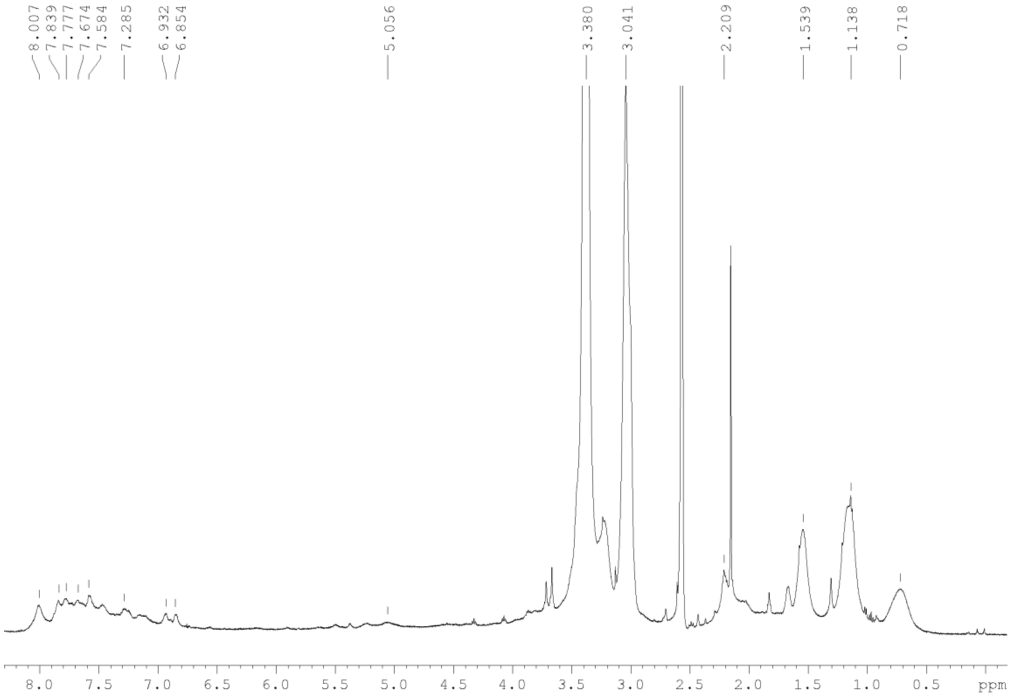

Figure S9. ^{1}H-NMR spectrum of **PAFAm** in DMSO-d_6.

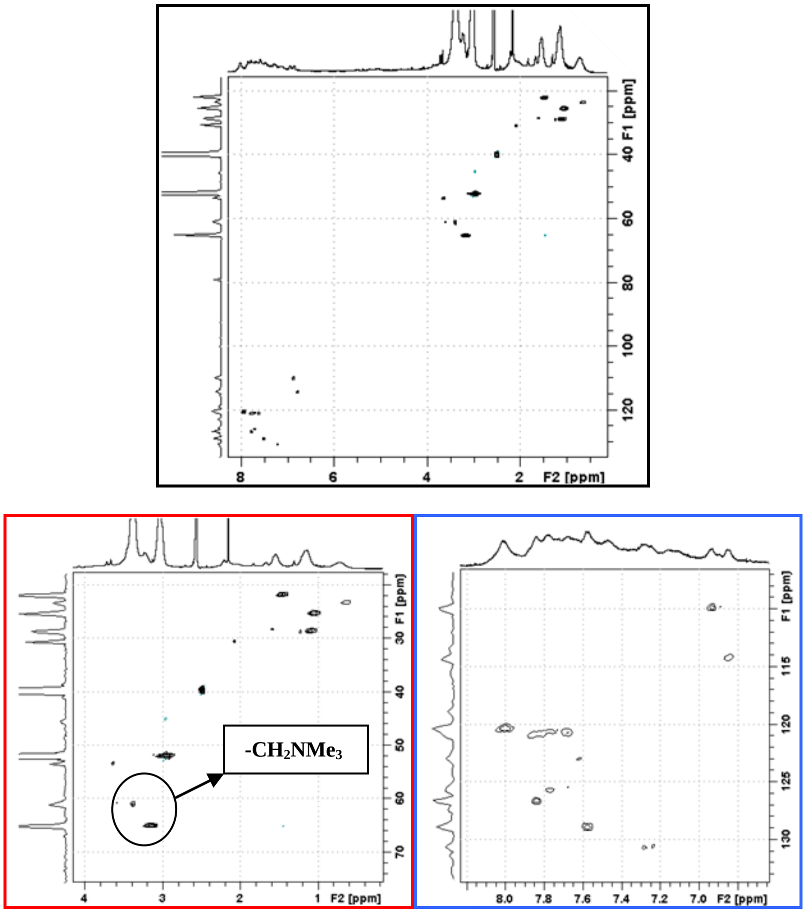

Figure S10. 2D ^{1}H-^{13}C **HMQC** NMR spectra of **PAFAm** in DMSO-d_6.

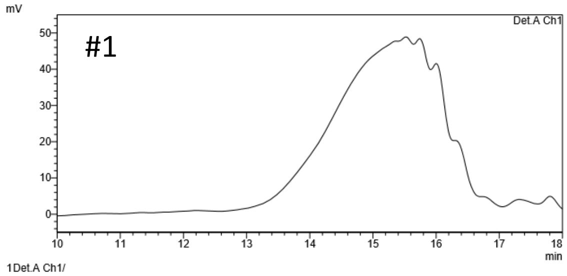

[Average Molecular Weight]

Number Average Molecular Weight(Mn)	5206
Weight Average Molecular Weight(Mw)	9904
Z Average Molecular Weight(Mz)	19568
Mw/Mn	1.90253

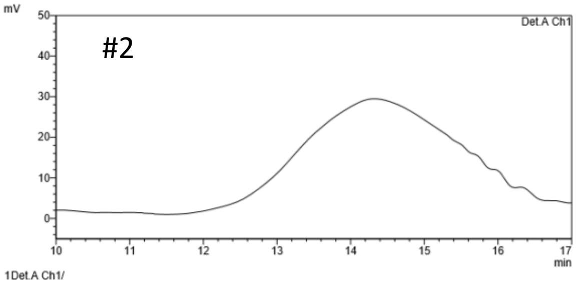

[Average Molecular Weight]

Number Average Molecular Weight(Mn)	12824
Weight Average Molecular Weight(Mw)	20773
Z Average Molecular Weight(Mz)	30605
Mw/Mn	1.61984

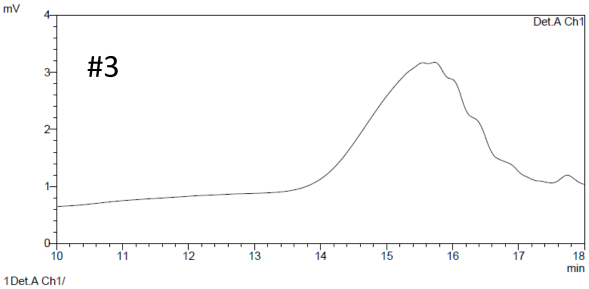

[Average Molecular Weight]

Number Average Molecular Weight(Mn)	3794
Weight Average Molecular Weight(Mw)	6393
Z Average Molecular Weight(Mz)	10513
Mw/Mn	1,68501

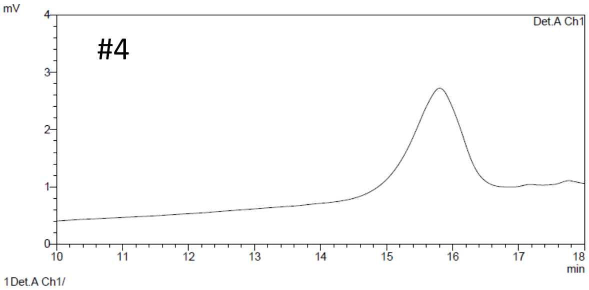

[Average Molecular Weight]

Number Average Molecular Weight(Mn)	3273
Weight Average Molecular Weight(Mw)	3543
Z Average Molecular Weight(Mz)	3823
Mw/Mn	1,08256

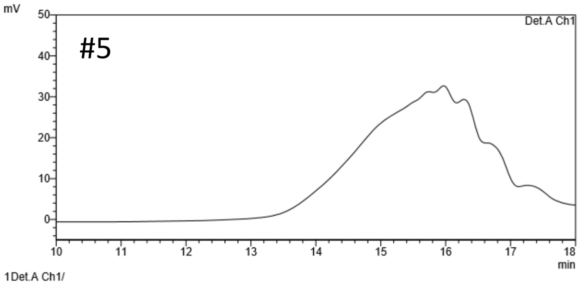

[Average Molecular Weight]

Number Average Molecular Weight(Mn)	3543
Weight Average Molecular Weight(Mw)	6909
Z Average Molecular Weight(Mz)	13366
Mw/Mn	1.94994

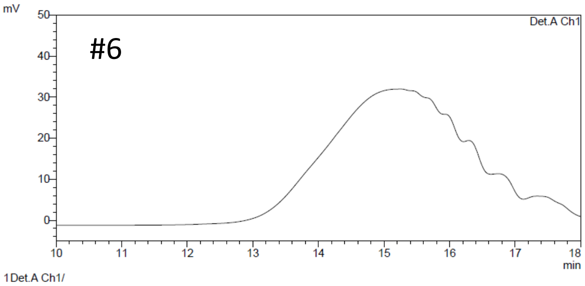

[Average Molecular Weight]

Number Average Molecular Weight(Mn)	6303
Weight Average Molecular Weight(Mw)	11803
Z Average Molecular Weight(Mz)	21099
Mw/Mn	1.87270

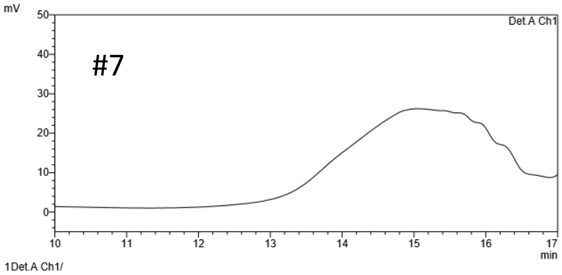

[Average Molecular Weight]

Number Average Molecular Weight(Mn)	5934
Weight Average Molecular Weight(Mw)	11601
Z Average Molecular Weight(Mz)	21991
Mw/Mn	1.95507

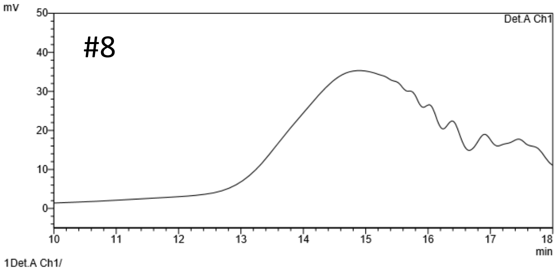

[Average Molecular Weight]

Number Average Molecular Weight(Mn)	7056
Weight Average Molecular Weight(Mw)	15018
Z Average Molecular Weight(Mz)	28747
Mw/Mn	2.12835

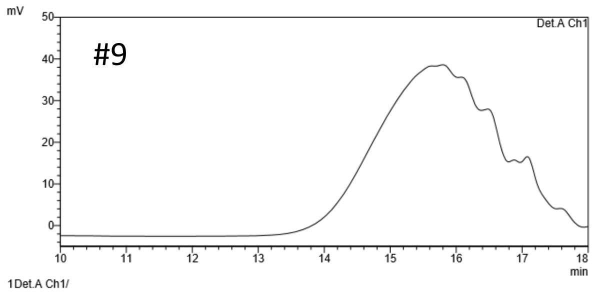

[Average Molecular Weight]

Number Average Molecular Weight(Mn)	2881
Weight Average Molecular Weight(Mw)	5427
Z Average Molecular Weight(Mz)	9212
Mw/Mn	1.88354

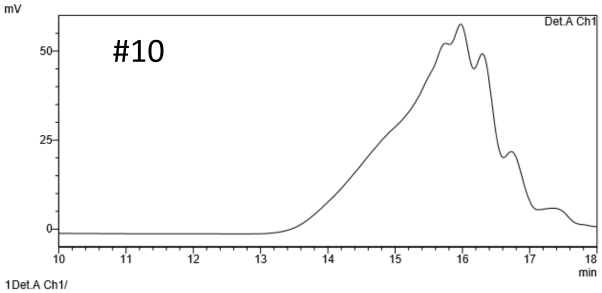

[Average Molecular Weight]

Number Average Molecular Weight(Mn)	3408
Weight Average Molecular Weight(Mw)	6256
Z Average Molecular Weight(Mz)	12068
Mw/Mn	1.83568

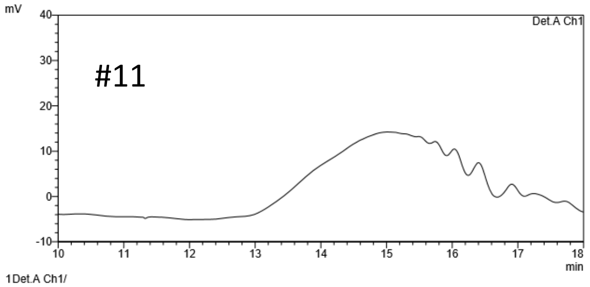

[Average Molecular Weight]

Number Average Molecular Weight(Mn)	5708
Weight Average Molecular Weight(Mw)	10700
Z Average Molecular Weight(Mz)	17997
Mw/Mn	1.87468

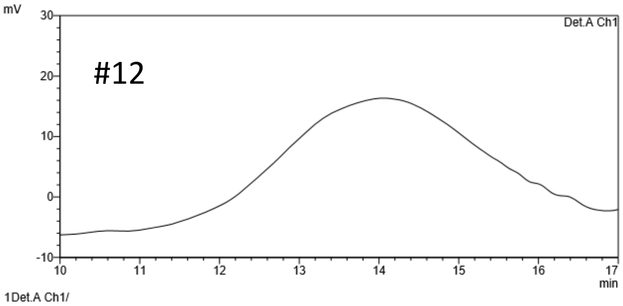

[Average Molecular Weight]

Number Average Molecular Weight(Mn)	13293
Weight Average Molecular Weight(Mw)	36486
Z Average Molecular Weight(Mz)	73633
Mw/Mn	2.74473

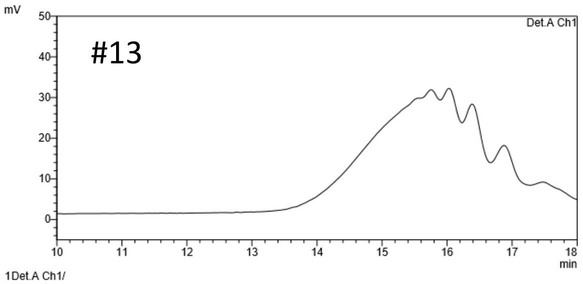

[Average Molecular Weight]

Number Average Molecular Weight(Mn)	3925
Weight Average Molecular Weight(Mw)	6446
Z Average Molecular Weight(Mz)	10995
Mw/Mn	1.64226

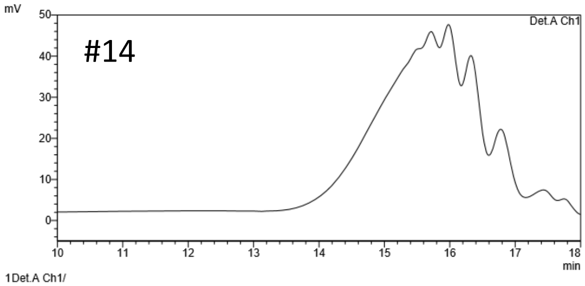

[Average Molecular Weight]

Number Average Molecular Weight(Mn)	3340
Weight Average Molecular Weight(Mw)	5734
Z Average Molecular Weight(Mz)	9931
Mw/Mn	1.71684

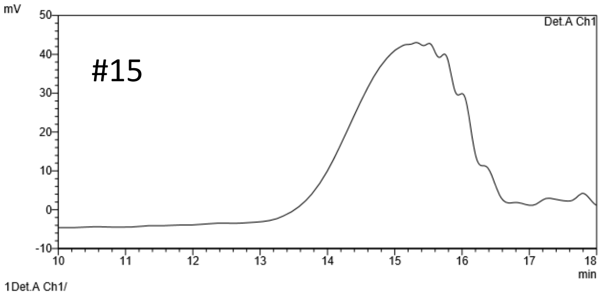

[Average Molecular Weight]

Number Average Molecular Weight(Mn)	5614
Weight Average Molecular Weight(Mw)	8862
Z Average Molecular Weight(Mz)	13589
Mw/Mn	1.57857

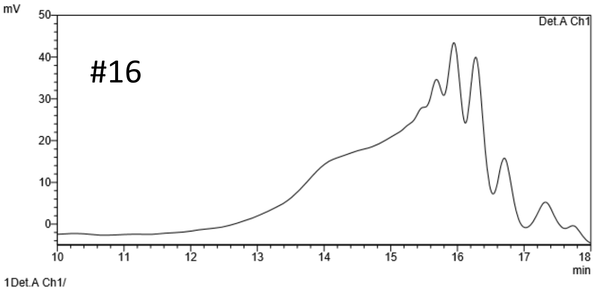

[Average Molecular Weight]

Number Average Molecular Weight(Mn)	4231
Weight Average Molecular Weight(Mw)	12769
Z Average Molecular Weight(Mz)	42529
Mw/Mn	3.01822

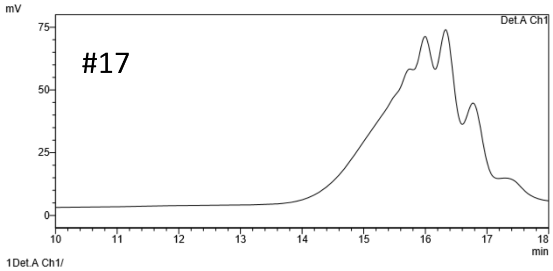

[Average Molecular Weight]

Number Average Molecular Weight(Mn)	2686
Weight Average Molecular Weight(Mw)	4210
Z Average Molecular Weight(Mz)	6937
Mw/Mn	1.56729

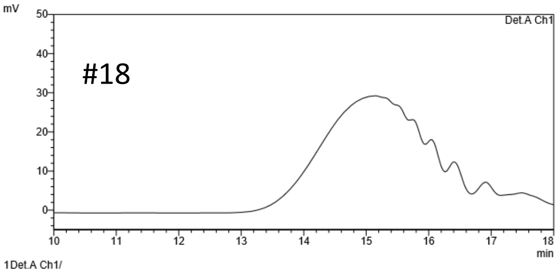

[Average Molecular Weight]

Number Average Molecular Weight(Mn)	5926
Weight Average Molecular Weight(Mw)	10109
Z Average Molecular Weight(Mz)	16182
Mw/Mn	1.70575

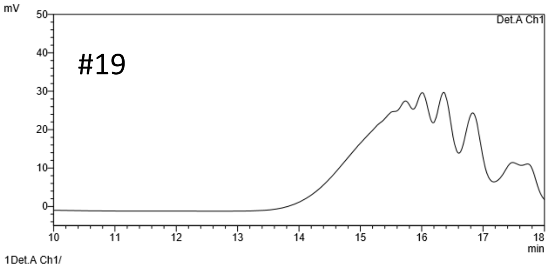

[Average Molecular Weight]

Number Average Molecular Weight(Mn)	2807
Weight Average Molecular Weight(Mw)	4883
Z Average Molecular Weight(Mz)	8225
Mw/Mn	1.73983

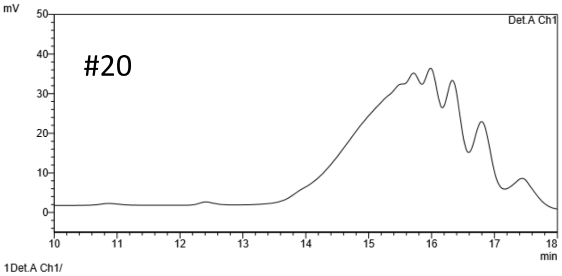

[Average Molecular Weight]

Number Average Molecular Weight(Mn)	3249
Weight Average Molecular Weight(Mw)	6082
Z Average Molecular Weight(Mz)	11273
Mw/Mn	1.87186

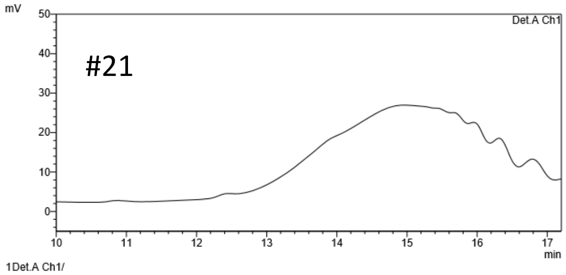

[Average Molecular Weight]

Number Average Molecular Weight(Mn)	5534
Weight Average Molecular Weight(Mw)	14140
Z Average Molecular Weight(Mz)	30027
Mw/Mn	2.55518

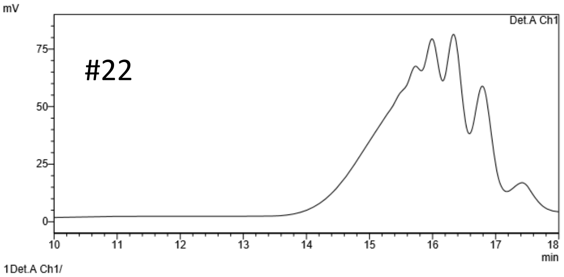

[Average Molecular Weight]

Number Average Molecular Weight(Mn)	2668
Weight Average Molecular Weight(Mw)	4390
Z Average Molecular Weight(Mz)	7442
Mw/Mn	1.64580

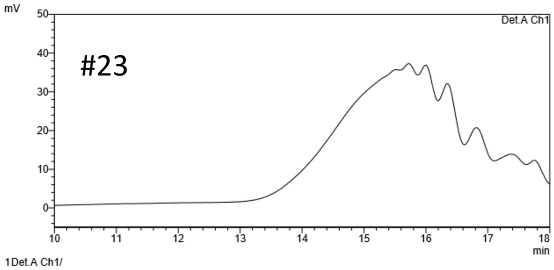

[Average Molecular Weight]

Number Average Molecular Weight(Mn)	3891
Weight Average Molecular Weight(Mw)	7420
Z Average Molecular Weight(Mz)	13703
Mw/Mn	1.90724

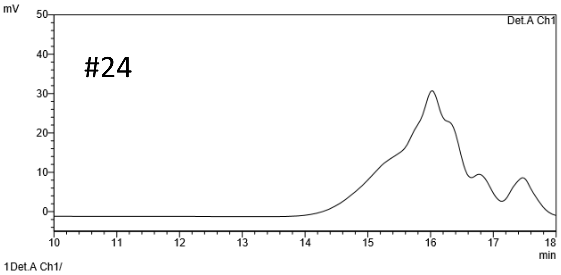

[Average Molecular Weight]

Number Average Molecular Weight(Mn)	2794
Weight Average Molecular Weight(Mw)	3956
Z Average Molecular Weight(Mz)	5761
Mw/Mn	1.41610

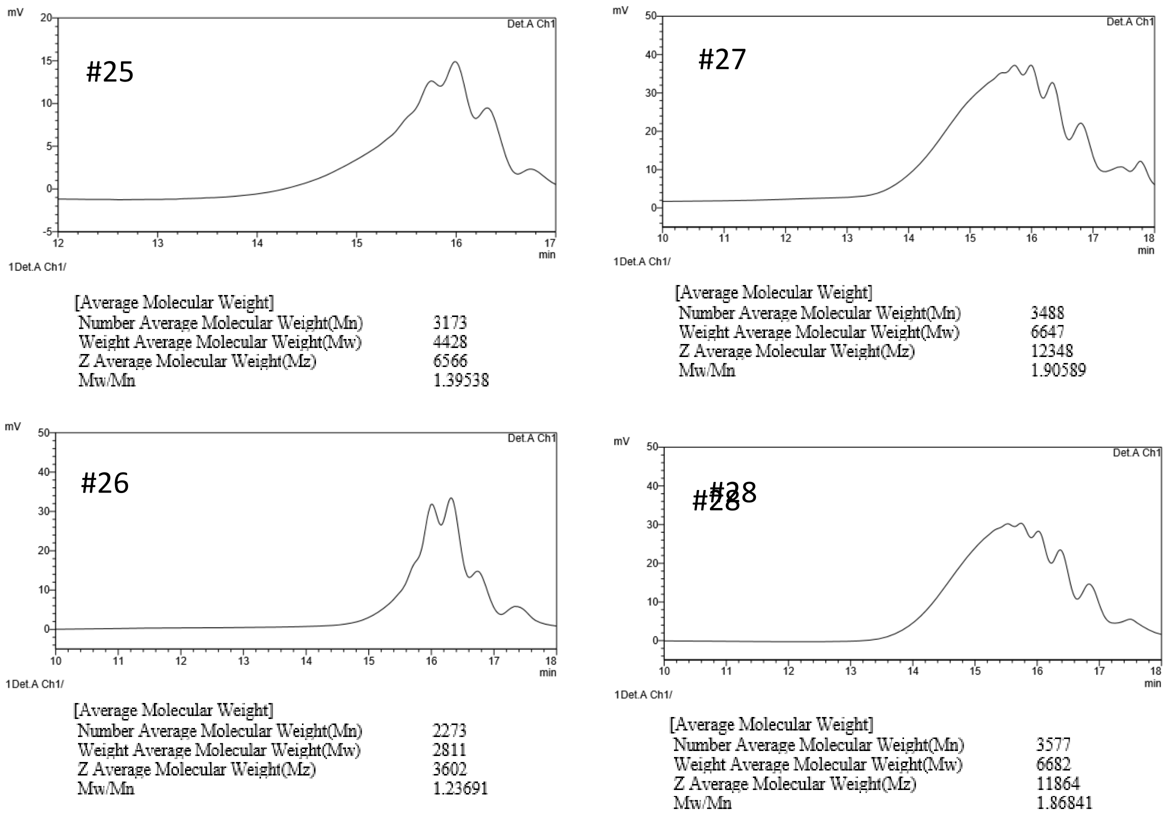

Figure S11. GPC Chromatograms of **PAF** batches.

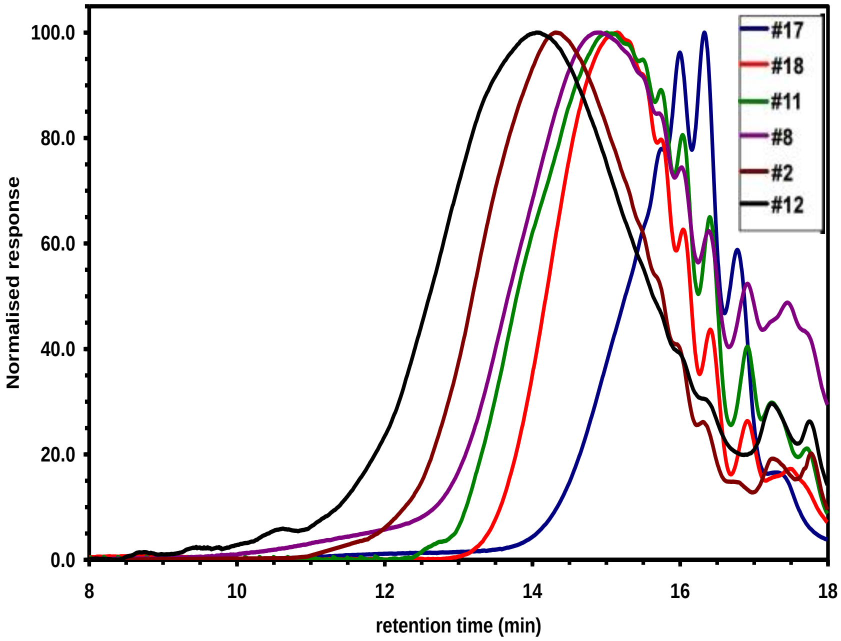

Figure S12. GPC Chromatograms for selected batches of **PAF** (Table S1).

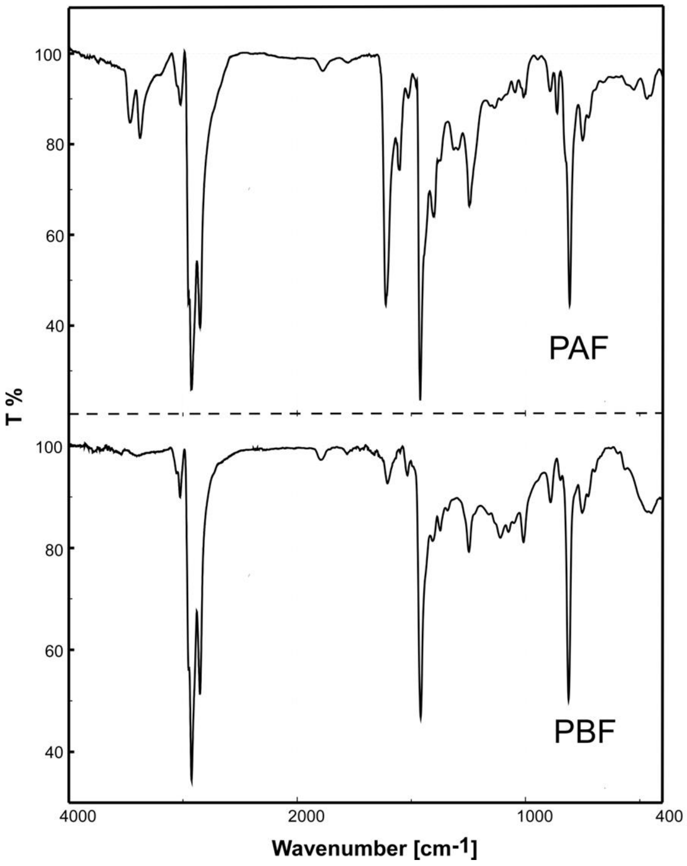

Figure S13. Comparative FT-IR spectra of **PAF** (top) and **PBF** (bottom) in BrK pellets.

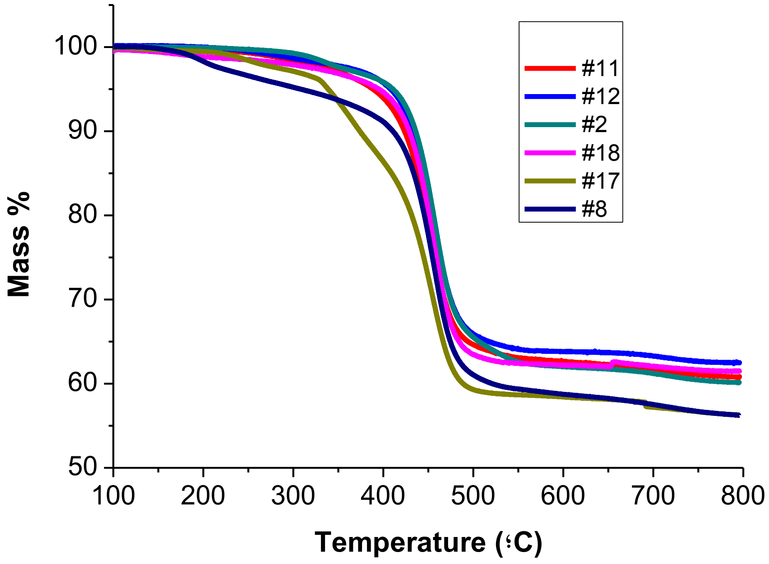

Figure S14. TGA curves for selected batches of **PAF** (Table S1).

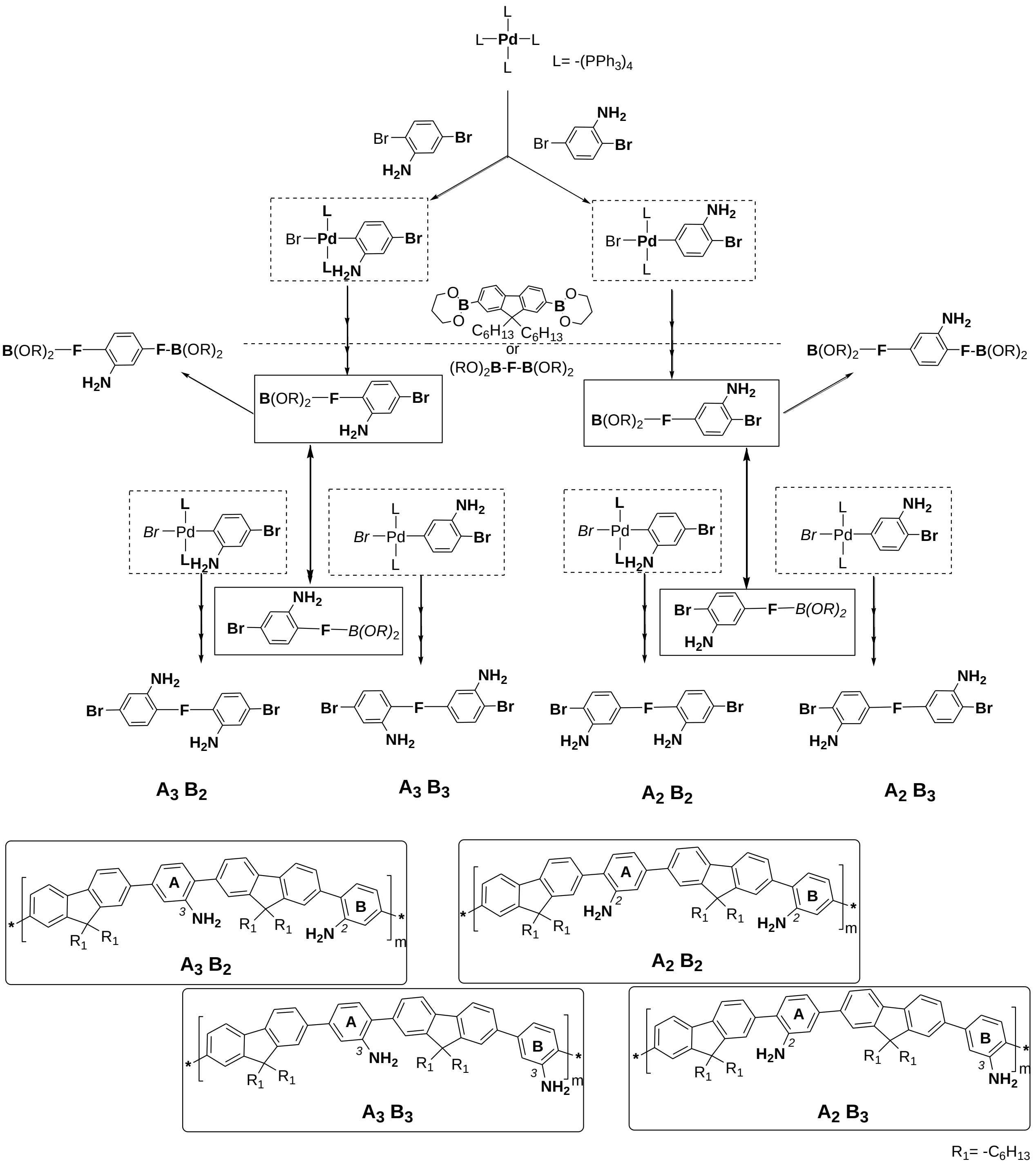

Figure S15. Proposed mechanism in the polymerization catalyzed by active species of palladium.

n= 8.........256........ 2:14:42:70:70:42:14:2

n= 9.........512........ 2:16:56:112:140:112:56:16:2

n= 10......1024........ 2:18:72:168:252:252:168:72:18:2

n= 8.........256........ 2:<u>14</u>:42:70:70:42:<u>14</u>:2

NUMBER DYADS: n-1

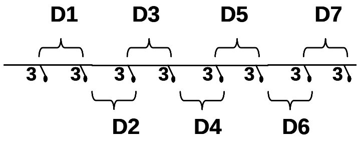

Analysis of probability for polymer sequence n=8 ,(2^8=256)

A. Two extreme cases of dyads (with two samples each):

- **Unidirectional Polymer**
  -Total number case: 2

Prediction NMR:
One peak higher 4.10ppm
Areas **(I:II); 100:0**

3,3,3,3,3,3,3,3 = **TH**/TH/TH/TH/TH/TH/TH

2,2,2,2,2,2,2,2 = **HT**/HT/HT/HT/HT/HT/HT

- **Alternating polymer**
  -Total number case: 2

Prediction NMR:
One peak lower 4.10ppm
Areas **(I:II); 0:100**

2,3,2,3,2,3,2,3 = **TT**/HH/TT/HH/TT/HH/TT

3,2,3,2,3,2,3,2 = **HH**/TT/HH/TT/HH/TT/HH

B. Two proximity extreme cases of dyads (with two samples each):

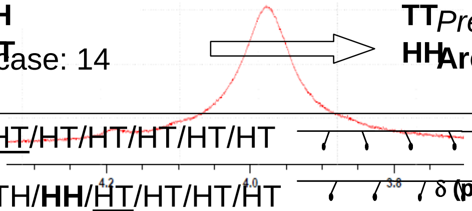

- **Block copolymer**
  -Total number case: 14

Prediction NMR: Two peaks
Areas (I:II) 6:1; % RR= 14%

2,3,3,3,3,3,3,3 = **TT**/HT/HT/HT/HT/HT/HT

2,2,**2,3**,3,3,3,3 = TH/TH/**HH**/HT/HT/HT/HT

- **"Quasi" alternating polymer**.
  -Total number case: 14

Prediction NMR:Two peaks
Areas (I:II) 1:6; %RR= 86%

2,3,**2,3**,3,2,3,2 = TT/HH/**TT**/HT/HH/TT/HH

3,2,**3,2**,2,3,2,3 = HH/TT/**HH**/TH/TT/HH/TT

TH/HT

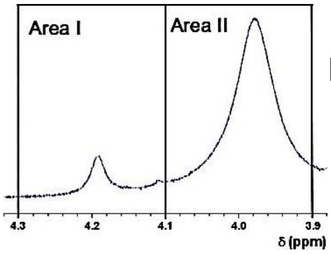

HH/TT

Figure S16. Analysis of probability for polymer sequence (n= number of chains) and correspondence of number of dyad cases, as normal distribution (number of cases: 2^n, for n= Mw/Munity).

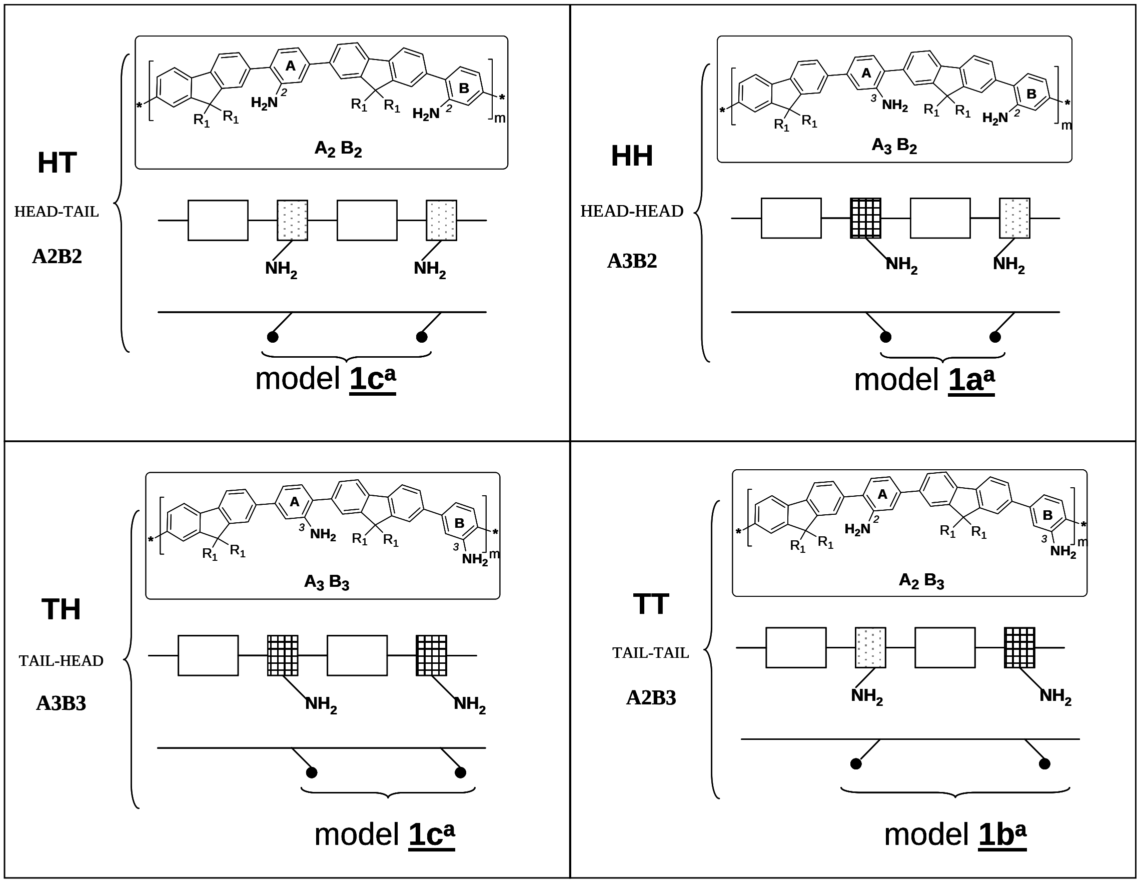

Figure S17. Symbols and structures used in this manuscript.

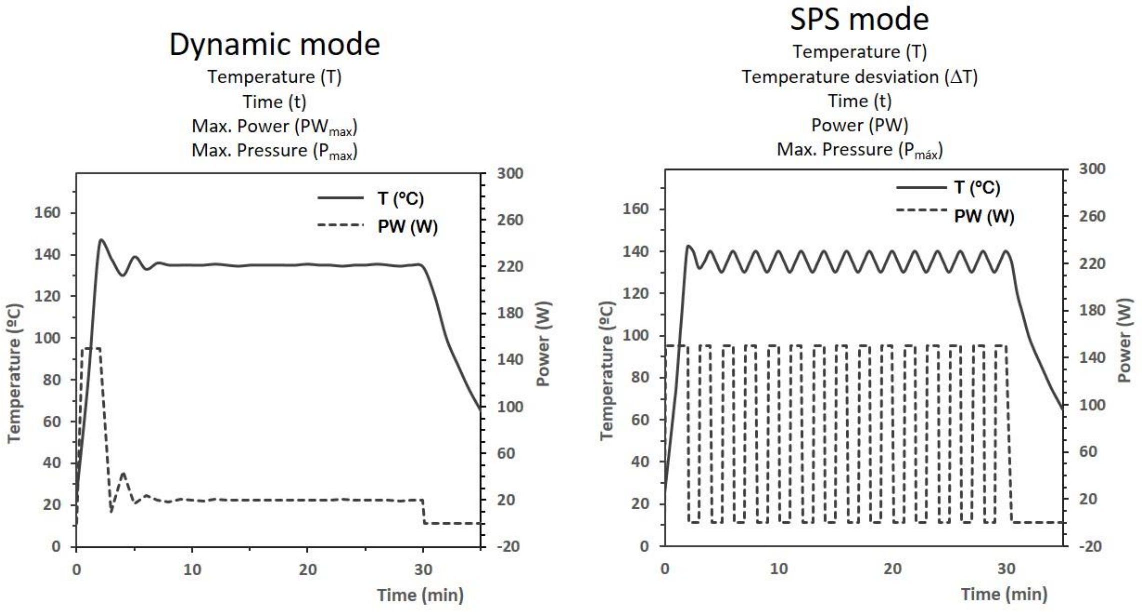

Figure S18. Temperature and power *versus* time for Suzuki Coupling microwave-assisted in Dynamic mode (left) and SPS mode, with temperature variation 5ºC (right).

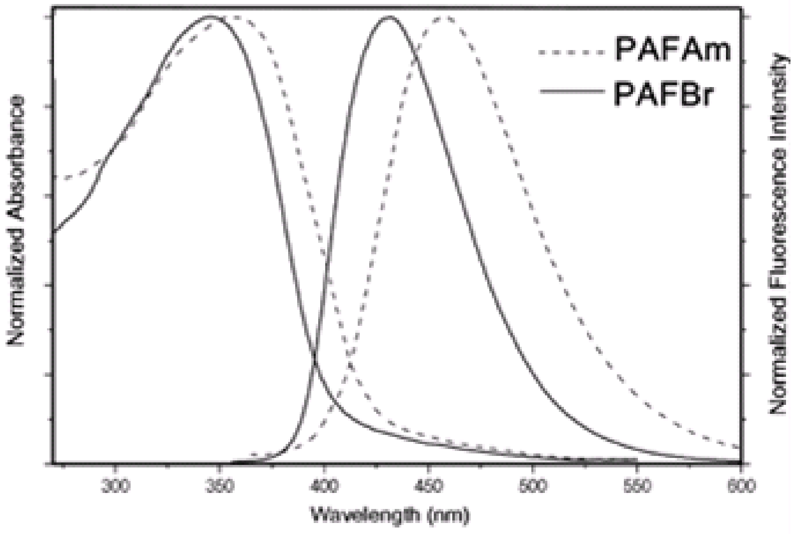

Figure S19. Normalized absorbance and emission spectra of PAFBr (solid line) and PAFAm (dashed line) in chloroform and water solutions, respectively.

Table S1. Optical data as function of RR of dyads in selected batches of **PAF.**

N #	Heating mode [a]	n [b]	% RR by NMR [c]	λ_{abs}^{max} (nm)	λ_{em}^{max} (nm)	$\Delta\lambda$ (nm)
17	MW-SPS	10	68	352	421	69
18	MW-SPS	23	95	360	419	59
11	Oil bath	24	93	360	421	61
8	MW-SPS	34	93	358	421	63
2	Oil bath	47	86	362	419	57
12	Oil bath	83	68	362	419	57
Ref[d]	Oil bath	82	-	361	422	61

a See experimental conditions of each batch.
b n (number of monomer unities) = M_w/M_u; where M_u = molecular weight unity (441.7 g/mol).
c. Percentages of regioregularity (%RR) were calculated in based on ratio NMR to integrated the Area II (3.9 > δ > 4.1 ppm) over Total Area (3.9 > δ > 4.3 ppm).
d. Yamaguchi, I.; Mizoguchi, N.; Sato, M. *Macromolecules* 2009, 42, (13), 4416-4425.

Table S2. Effect of polymerization time.

N #	Heating mode [b]	Time	Yield (%)	M_w [a] (kg/mol)	PDI [b]	n [b]	% RR by NMR [c]
1	Oil bath	1 d	62	9.90	1.9	22	75
15		2 d	55	8.86	1.6	20	75
2		3 d	86	20.77	1.6	47	86
16		4 d	58	10.70	1.9	24	61
17	SPS	7 min	37	4.21	1.6	10	68
7		14 min	90	11.60	2.0	26	72
8		22 min	99	15.02	2.1	34	93
18		30 min	93	10.11	1.7	23	95

a. Mw= weight-average molecular weight, estimated by GPC in THF on the basis of polystyrene calibration.
b. PDI (Polydispersity index) = M_w/Mn; where Mn = number-average molecular weight, and n (number of monomer unities) = M_w/M_u; where M_u = molecular weight unity (441.7 g/mol).
c. Percentages of regioregularity (%RR) were calculated in based on ratio NMR to integrated the Area II (3.9 > δ > 4.1 ppm) over Total Area (3.9 > δ > 4.3 ppm).

Table S3. Effect of microwave parameter: solvent and volume on the polymerization.[a]

N #	Solvent (v/v)	Volume (mL)	Yield (%)	M_w (kg/mol)	PDI	n	% RR by NMR [c]
19	THF/H_2O (2:1)	6	96	4.88	1.7	11	60
7	$Toluene/H_2O$ (2:1)	6	90	11.60	2.0	26	72
20		4.5	73	6.08	1.9	14	77
21		3	86	14.14	2.6	32	82

a Same description on table S1.

Table S4. Effect of power microwave on the polymerization.[a]

N #	Power (W)	Yield (%)	M_w (kg/mol)	PDI	n	% RR by NMR
22	100	56	4.39	1.7	10	70
7	150	90	11.60	2.0	26	72
23	200	84	7.42	1.9	17	81

a Same description on table S1.

Table S5. Effect of reaction temperature and the temperature fluctuation on the polymerization.[a]

N #	Temp. (ºC)	ΔT (ºC)	Yield (%)	M_w (kg/mol)	PDI	n	% RR by NMR
24	80	1	3	3.96	1.4	9	38
25		5	5	4.43	1.4	10	64
26	100	5	11	2.81	1.3	6	51
27	135	1	72	6.65	1.9	15	78
7		5	90	11.60	2.0	26	72
28	150	5	86	6.68	1.9	15	84

a Same description on table 1.
